# Current Application of Advancing Spectroscopy Techniques in Food Analysis: Data Handling with Chemometric Approaches

**DOI:** 10.3390/foods12142753

**Published:** 2023-07-19

**Authors:** Mourad Kharbach, Mohammed Alaoui Mansouri, Mohammed Taabouz, Huiwen Yu

**Affiliations:** 1 Department of Food and Nutrition, University of Helsinki, 00014 Helsinki, Finland; 2 Department of Computer Sciences, University of Helsinki, 00560 Helsinki, Finland; 3 Nano and Molecular Systems Research Unit, University of Oulu, 90014 Oulu, Finland; 4 Research Unit of Mathematical Sciences, University of Oulu, 90014 Oulu, Finland; 5 Biopharmaceutical and Toxicological Analysis Research Team, Laboratory of Pharmacology and Toxicology, Faculty of Medicine and Pharmacy, University Mohammed V in Rabat, Rabat BP 6203, Morocco; 6 Shenzhen Hospital, Southern Medical University, Shenzhen 518005, China; 7 Chemometrics group, Faculty of Science, University of Copenhagen, Rolighedsvej 26, 1958 Frederiksberg, Denmark

**Keywords:** food analysis, food authenticity, food chemicals, spectroscopy techniques, chemometrics, multivariate analysis

## Abstract

In today’s era of increased food consumption, consumers have become more demanding in terms of safety and the quality of products they consume. As a result, food authorities are closely monitoring the food industry to ensure that products meet the required standards of quality. The analysis of food properties encompasses various aspects, including chemical and physical descriptions, sensory assessments, authenticity, traceability, processing, crop production, storage conditions, and microbial and contaminant levels. Traditionally, the analysis of food properties has relied on conventional analytical techniques. However, these methods often involve destructive processes, which are laborious, time-consuming, expensive, and environmentally harmful. In contrast, advanced spectroscopic techniques offer a promising alternative. Spectroscopic methods such as hyperspectral and multispectral imaging, NMR, Raman, IR, UV, visible, fluorescence, and X-ray-based methods provide rapid, non-destructive, cost-effective, and environmentally friendly means of food analysis. Nevertheless, interpreting spectroscopy data, whether in the form of signals (fingerprints) or images, can be complex without the assistance of statistical and innovative chemometric approaches. These approaches involve various steps such as pre-processing, exploratory analysis, variable selection, regression, classification, and data integration. They are essential for extracting relevant information and effectively handling the complexity of spectroscopic data. This review aims to address, discuss, and examine recent studies on advanced spectroscopic techniques and chemometric tools in the context of food product applications and analysis trends. Furthermore, it focuses on the practical aspects of spectral data handling, model construction, data interpretation, and the general utilization of statistical and chemometric methods for both qualitative and quantitative analysis. By exploring the advancements in spectroscopic techniques and their integration with chemometric tools, this review provides valuable insights into the potential applications and future directions of these analytical approaches in the food industry. It emphasizes the importance of efficient data handling, model development, and practical implementation of statistical and chemometric methods in the field of food analysis.

## 1. Introduction

The growing world population is increasing the demand for food in multiple ways, which is leading to a higher demand for safety and quality control of commercialized products. Food can become contaminated by chemicals and physical substances through accidental or intentional means. In recent years, there have been several major cases of food adulteration, highlighting the critical need for controlling product authentication [1]. In one instance, wheat gluten samples were infused with melamine to improve their protein content. In 2008, China experienced a milk scandal where milk was found to be adulterated with melamine, and in 2012, India had a similar scandal where milk was found to be adulterated with detergent, urea, and other substances [2]. Several noteworthy incidents occurred in 2005, including the adulteration of chili powder with dye and the contaminated chili powder in Indian Worcestershire sauce [3]. Gelatin-like chemicals were used in the aquaculture market recently to increase weight in many instances in China. Spices are frequently adulterated with ground material worldwide, particularly in Europe and India [4,5]. These incidents demonstrate the importance of product authentication and quality control in the food industry to protect consumers from harmful and potentially dangerous adulterated products. To gain economic benefit, consumers are at risk of being exposed to serious health threats if food products are adulterated with cheap or chemical materials. This was seen in the Chinese milk scandal, where six infants passed and several thousand were hospitalized. A further case is the contamination of paprika with lead oxide to give it a reddish color, which caused over sixty hospitalized. In recent years, both controlling agencies and the public have grown increasingly concerned about the application of the phthalate plasticizer di-2-ethylhexyl phthalate as a clouding agent in beverages and food [6,7]. In the past decade, both food and feed products have been found to be adulterated. Some of the most adulterated agro-food commodities include honey, edible oils, and spices. Additionally, it has been reported that food products such as milk products, fruit extract, flour, coffee, alcohol, and meats, are being adulterated more frequently [8]. All these examples illustrate a significant global problem that poses a threat to consumers and has prompted food authorities to increase their scrutiny and inspection of the food production chain from farming to consumption.

Food adulteration can occur for various reasons, including complex production processes and long supply chains. Therefore, authentication is crucial for both labeling organizations and industries that must test raw materials and finished products to ensure compliance with specifications [9]. Additionally, confirming the authenticity of the food is essential for maintaining quality and preventing economic fraud. To address authentication challenges and ensure product quality, fast, reliable, and competent analytical methods are needed. The detection of composition properties and contaminants in food and agricultural commodities can be accomplished using a wide variety of physical and chemical methods. These properties, such as density, texture, color, acidity, and solubility, are typically measured by physical or chemical methods. The chemical composition of the samples determines the different chemical techniques involved and used in the identification of components and contamination detection of food commodities [10]. Despite being powerful analytical techniques, separation techniques including liquid-chromatography or gas-chromatography are not always suitable when the workflow is rapid and the experiments are costly, as the samples may be damaged during the study process. Therefore, advanced spectroscopic techniques, along with chemometrics, are now being used for the quality analysis and authentication of a wide range of food products. The main advantages of these techniques are that they are non-damaging, rapid, ecologically friendly, and economical. Spectroscopy data are complex and are typically handled by chemometric approaches for supervised and unsupervised pattern recognition, such as hierarchical cluster analysis (HCA), principal component analysis (PCA), linear discriminant analysis (LDA), soft independent modeling of class analogy (SIMCA), and partial least squares-discriminant analysis (PLS-DA) among others. These tools are mainly used to assess classes, such as attributing samples as either adulterated/unadulterated or authentic/not authentic. In addition to the qualitative chemometric approaches, there are also quantitative approaches including multivariate calibration tools, including principal component regression (PCR), and partial least squares (PLS) [11,12]. These tools are mainly used for quantitative parameters to quantify the number of adulterants and fatty acids content, based on data generated from spectroscopic techniques.

The focus of this review is to provide an overview of recent studies on advanced spectroscopy techniques and chemometric methods that are widely utilized in food evaluation, safety assessment, quality analysis, and manufacturing processes. The review aims to emphasize the advantages of these techniques over traditional analytical methods and underscore the importance of efficient data handling, model construction, and practical implementation of statistical and chemometric approaches in the field of food analysis. Additionally, the review aims to discuss the potential applications and future directions of these techniques in the food industry, while addressing the challenges associated with traditional analytical methods, including their destructiveness, laborious nature, time consumption, cost, and negative environmental impact.

## 2. Chemometric Approaches in Spectroscopy Data

**Figure 1:**
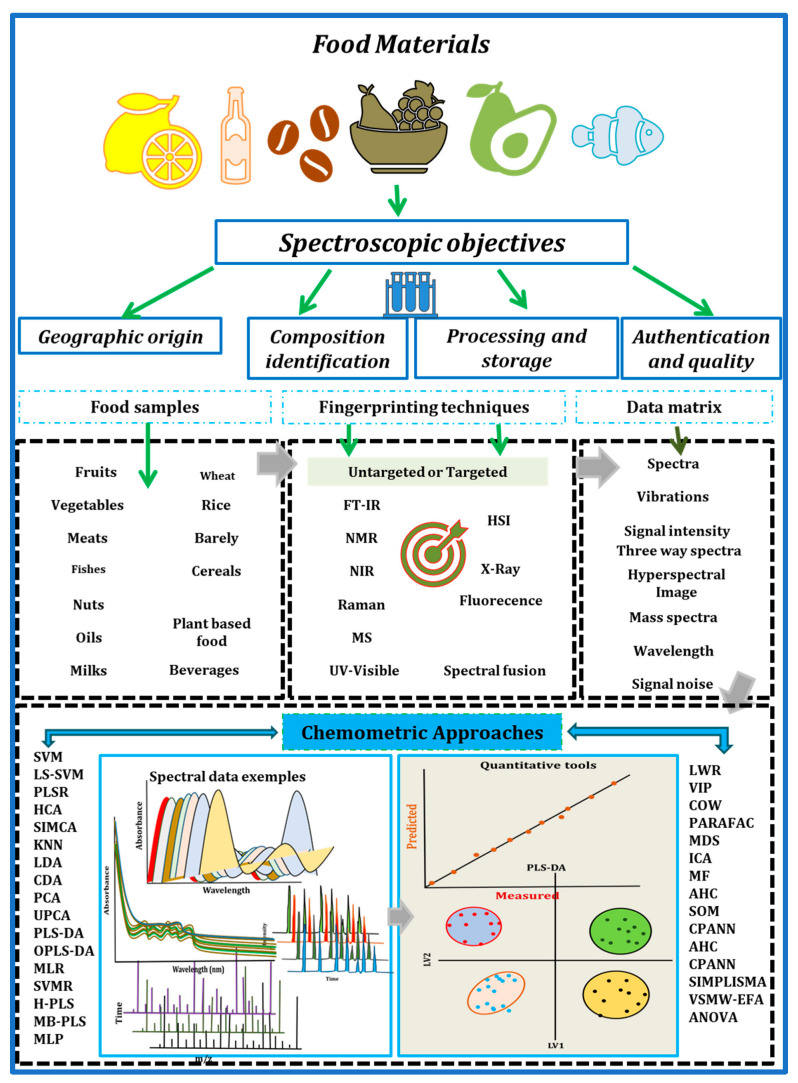
A general workflow of advanced spectroscopic techniques combined with chemometric approaches for food analysis.

**Figure 2:**
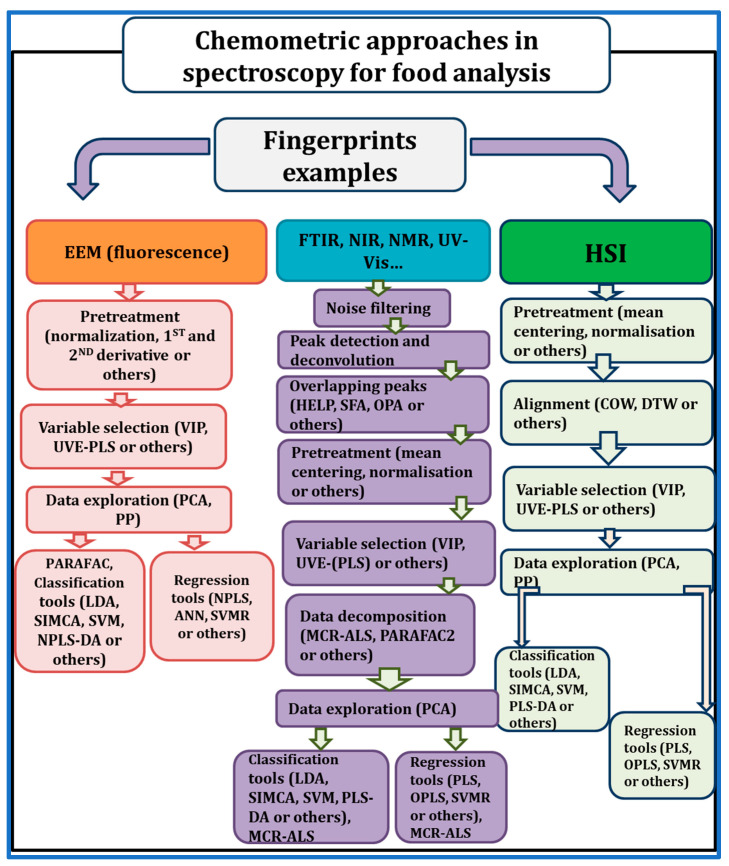
A roadmap example for chemometric approaches applied to different spectroscopic data for both quantitative and qualitative food analysis purposes.

The advancement of modern instruments, represented by spectroscopy techniques, has accelerated the development of the food industry and food research in recent decades. As a result, the data available to food analysts has become increasingly complex. Not only does the amount of data tend to be large, but the dimensionality of the data can also increase dramatically. Effectively analyzing and managing large amounts of spectroscopy data from food production, food processing, and food research is both a practical and theoretical issue. Chemometrics, which combines the power of statistics and mathematics for use by chemists, provides a valuable solution to the challenging analytical issues in food spectroscopy analysis. The general framework and pipeline of advanced spectroscopic techniques coupled with chemometric approaches applied in food analysis is illustrated in Figure 1. Advanced spectroscopic techniques combined with chemometric approaches are of great significance in food analysis. These techniques provide fast, precise, and non-destructive measurements of diverse food properties (Figure 1). Their integration offers multiple advantages, such as rapid analysis, non-destructive measurement, multivariate data analysis, quality control, process optimization, and allergen detection. These advancements contribute significantly to enhancing food safety, ensuring quality assurance, and safeguarding consumer protection. On the other hand, several chemometric tools have been developed and validated to be powerful in terms of information extraction, multivariate relationship analysis, prediction, and discrimination analysis, among others, in food spectroscopy data analytics. A roadmap workflow example in Figure 2 for using chemometric tools to handle spectral data in different scenarios for either qualitative or quantitative purposes. However, the interpretation of spectroscopy data, be it in the form of signals or images, can be intricate without the aid of statistical and innovative chemometric approaches. These approaches encompass crucial steps, including pre-processing, exploratory analysis, variable selection, regression, classification, and data integration. By employing these methods, researchers can extract pertinent information and effectively manage the complexities inherent in spectroscopic data.

### 2.1. Pre-Processing Techniques

Pre-processing plays a crucial role in spectral data analysis as it prepares the data for further analysis and modeling. Various pre-processing techniques are employed to address common challenges such as scattering correction, baseline correction, peak shift alignment, denoising, and handling missing values. Effective pre-processing is essential for enhancing the performance of models by eliminating artifacts from the data and reducing fitting errors. By applying appropriate pre-processing methods, the spectral data becomes more reliable and conducive to accurate analysis and interpretation. In the context of missing data, several chemometric tools have been developed to address this issue [13]. For instance, maximum likelihood PCA-based imputation [14], as well as data regression methods like KDR-PLSR (kernel density-ratio-based partial least squares regression) and KDR-PCR (kernel density-ratio-based principal components regression) [14], have recently emerged as highly effective approaches for handling missing data. For multi-way data, such as fluorescence data, practical solutions for missing data imputation include alternating least squares with single imputation, Parallel Factor (PARAFAC) analysis, and the Levenberg-Marquardt method [15,16]. These techniques offer reliable and efficient means to impute missing data, enabling more comprehensive and accurate analyses of complex datasets. In the context of scatter correction, Multiplicative Scatter Correction (MSC) [17], Standard Normal Variate (SNV) [18], and other normalization methods are widely employed chemometrics techniques. These methods effectively address the issue of scatter in spectral data. Additionally, advancements in scatter correction tools have led to the development of new versions and improved approaches [17]. To tackle baseline drift problems in spectral data, adaptive reweighing schemes for polynomial fitting and penalized least squares [19], as well as Tikhonov regularization [20], have proven successful in removing unwanted baseline variations. These techniques offer reliable means to address baseline drift and enhance the accuracy of subsequent analysis.

In order to mitigate peak shift problems, several methods have been reported to be useful. Automatic time shift alignment (ATSA) [21], coherent point drift peak alignment [22], Global peak alignment with point matching algorithm [23], and PARAFAC Applied to Shift Invariant Amplitude Spectra (PARASIAS) [24] are among the techniques that have demonstrated effectiveness in aligning peaks accurately despite shifts or distortions. Derivative calculation of spectral data is a promising solution to some different artefacts problems especially when it is combined with other techniques, e.g., combining the first derivative and simple spectral ratio can correct both the additive effects and multiplicative effects [25].

Overall, the utilization of these advanced techniques in scatter correction, baseline drift correction, and peak shift alignment significantly improve the quality and reliability of spectral data, facilitating more robust and accurate analyses in various chemometric applications.

### 2.2. Variable Selection Tools

Variable selection is a valuable technique in spectral data analysis as it enhances model performance, provides better interpretations, and reduces measurement costs [26]. Several popular methods are commonly used in chemometrics for spectral data analysis, including model factors and assessment, model-based feature importance statistics, interval partial least squares regression (iPLS) [27], and genetic algorithm (GA) [28]. When considering model parameters, variables with lower loadings and regression coefficients may not be as important as those with higher values. In the case of model-based variable importance statistics, variables such as variables important for projection (VIP) [29], which measures the contribution of a variable in describing the data, and selectivity ratios [30], which evaluate the predictive performance of variables, are commonly used in chemometrics analysis of spectral data. These statistics help identify the most influential variables in the model. The iPLS is a variable selection method that operates by selecting windows of variables, making the selection of window size a critical aspect of iPLS. On the other hand, GA performs variable selection by simulating the process of natural selection, estimating models involving patterns in variable generation [26]. However, GA requires more complex parameter settings compared to other variable selection methods. Regardless of the specific variable selection method employed, it is important to remember that validation is always necessary to avoid erroneous conclusions resulting from overfitting. Validation procedures help ensure the robustness and reliability of the selected variables and the overall model performance.

In summary, the utilization of variable selection techniques in spectral data analysis offers improved model performance, interpretability, and cost-effectiveness. Methods such as iPLS, GA, and model-based feature importance statistics contribute to selecting relevant variables and enhancing the accuracy and reliability of chemometric analyses. Validation procedures are crucial to validate the selected variables and mitigate the risk of overfitting.

### 2.3. Exploratory and Clustering Tools

Exploratory data analysis is a critical component of food spectroscopy data analysis, serving as a means to understand descriptive statistical characteristics and gain multivariate insights from complex datasets. A range of exploratory analysis tools, including both graphical techniques and quantitative dimensionality reduction techniques, are widely utilized in the analysis of food spectroscopy data. Graphical techniques such as box plots, histograms, and scatter plots are valuable for visualizing the distribution, variation, and presence of missing values in samples. These tools are commonly employed in the analysis of metabolite data obtained from mass spectroscopy [31]. By utilizing graphical tools, researchers can effectively explore the properties and patterns present within the data.

Principal Component Analysis (PCA) is a representative dimensionality reduction technique and one of the most popular tools employed in food spectroscopy data analysis. The earliest invention of PCA dates back to the early 20th century [32]. However, this first invention focused more on the modeling property and explained the variation of PCA. However, it was later expanded upon by Hotelling, who introduced the concept of PCA as a linear combination of variables [33]. Nowadays, PCA is now widely recognized for its ability to reveal complex relationships within multivariate data, making it a powerful tool for obtaining an overview of complex datasets. It is frequently used to explore relationships between samples and variables, identify outliers, discover and determine patterns (groups), as well as generating new hypotheses [34]. Moreover, PCA can also be utilized for conducting clustering analysis. By examining the relationships between samples, PCA can effectively divide samples with different statistical characteristics into distinct groups. In addition to PCA, Hierarchical Cluster Analysis (HCA) [35] and k-means clustering analysis [36] are widely used as clustering analysis tools in food spectroscopy data analysis. In the format of a dendrogram, HCA constructs a dendrogram that hierarchically divides samples into groups based on their similarities, facilitating the identification of distinct clusters. On the other hand, K-means clustering analysis partitions objects into k-clusters, with each object belonging to the group with the closest average.

Overall, exploratory data analysis, comprising graphical techniques, dimensionality reduction methods like PCA, and clustering analysis tools such as HCA and k-means, enables researchers to gain insights into complex food spectroscopy datasets. These approaches facilitate the understanding of data properties, identification of patterns, and generation of hypotheses, ultimately advancing the knowledge and interpretation of food analysis.

### 2.4. Regression and Prediction Tools

Prediction models are essential in food spectroscopy data analysis, as they enable the successful prediction of chemical and physical properties, supporting green food research and the sustainable food industry. Partial least squares (PLS) regression is a standard chemometric method used for prediction analysis of spectral data [37]. By projecting the predictor variables and response variables onto a new space, PLS seeks to uncover the underlying relationship between X and Y matrices by modeling the covariance structures in this new space [38]. PLS has the added advantage of effectively handling the collinearity problem often encountered in spectral data [39]. In addition to PLS, N-way PLS [40] is a powerful tool for handling multi-way spectral data and the N-way PLS model is advantageous in well modeling performance, robustness to noise, stabilized solution, and improved prediction capabilities [41] specifically for multi-way spectral data. Various other regression methods, such as support vector machine (SVM) regression artificial neural network (ANN) and multiple linear regression (MLR), are also employed in spectral data analysis [42]. MLR is a linear regression method that establishes the relationship between independent variables and dependent variables by fitting a linear model. However, MLR has strict assumptions that need to be met, including constant variance of the residuals, multivariate normality, and linear assumption [43], which can limit its applications in certain cases. In contrast, SVM regression and ANN are often utilized for non-linear prediction analysis in spectral data. For instance, ANN can capture highly non-linear relationships between inputs and outputs, enabling the prediction of an output variable based on input data [44]. The non-linear models such as SVM and ANN can offer attractive predictive performance in many applications [42], but the issue of overfitting must be carefully addressed. Therefore, validation is a crucial step in non-linear prediction analysis to ensure the reliability and generalization of the models when applied to spectral data.

In short, prediction models, including PLS regression, N-way PLS, SVM regression, ANN, and MLR, are valuable tools in food spectroscopy data analysis. These models enable the prediction of chemical and physical properties, and their selection depends on the nature of the data, linearity assumptions, and the need for handling collinearity or capturing non-linear relationships. Validation procedures are essential to evaluate and validate the performance of these models, ensuring their accuracy and robustness in spectral data analysis.

### 2.5. Classification Tools

Classification is an essential task in food analysis, aimed at identifying and assigning categories to samples in order to detect pattern differences and enable subsequent analysis. Various chemometrics techniques are employed for the classification analysis in food analysis, including tree-based methods, regression-based methods, discriminant analysis, and neural networks, among others. One popularly used classification tool in food analysis is partial least squares-discriminant analysis (PLS-DA) [45]. PLS-DA is a discrimination method based on PLS regression but incorporates an additional classification step based on the thresholding of predicted y-values. Another method, the soft independent modelling class analogy (SIMCA) method combines the concept of PCA in performing classification analysis [46]. SIMCA utilizes the residuals from disjoint PCA models to assign samples to one or several classes, with the critical distance being based on the F-distribution [47]. In addition to PLS-DA and SIMCA models, Linear Discriminant Analysis (LDA) [48] and SVM [49] are also frequently used for classification analysis in food analysis. LDA models the differences between groups by finding a linear combination of features and projecting them from a higher dimensional space into a lower dimensional space, effectively separating them into distinct classes. On the other hand, SVM can also be used for non-linear classification analysis. In the case of non-linear classification, SVM employs a kernel function to transform the data from a non-linear space to a linear space, enabling the classification task in a high-dimensional space. However, it’s important to note that performing SVM classification on large spectroscopy datasets may be time-consuming due to the training and kernel computation requirements.

In brief, classification is a fundamental data analysis task in food analysis, and several chemometric techniques such as PLS-DA, SIMCA, LDA, and SVM are commonly utilized for this purpose. These methods enable the identification of patterns and the assignment of samples to different categories, facilitating subsequent analysis and decision-making in the field of food analysis. It’s important to consider the nature of the data, linear or non-linear relationships, and computational considerations when selecting the appropriate classification method for a given analysis.

### 2.6. Mixture Analysis Tools

Mixture analysis is a critical area of focus in food analysis, driven by advancements in omics technologies and the demand for improved production processes in the food industry. A wide range of chemometric tools is employed to analyze mixtures in food, encompassing both two-way analytical methods and multi-way analytical methods.

Multivariate curve resolution-alternating least squares (MCR-ALS) is one of the representative two-way chemometrics tools used for food mixture analysis. It allows for the extraction of chemically meaningful bilinear models from a data matrix that includes mixed measurements, with an additive model structure [50]. Basically, MCR-ALS decomposes the mixture data matrix into two matrices and a residual matrix. For instance, in the case of high-performance liquid chromatography with diode array detection (HPLC-DAD) data, MCR-ALS separates the data into a matrix containing elution profiles of all components, another matrix containing the corresponding pure spectra, and a residual matrix of the same dimensions as the raw data, capturing unexplained variations. Although MCR-ALS encounters challenges such as permutation ambiguity, intensity ambiguity, and rotational ambiguity, these issues can be partially addressed through specific strategies [51]. Parallel Factor Analysis (PARAFAC) [52] and Parallel Factor Analysis2 (PARAFAC2) [53] models are widely used multi-way models for complex mixture analysis. These models generalize the bilinear models to handle multi-way data. Instead of generating a set of bilinear components, PARAFAC and PARAFAC2 decompose the high-order tensor data into a set of trilinear components (in the case of three-way data) in which each vector represents the information from each mode. Due to the unique advantages of PARAFAC and PARAFAC2 models [54], they are powerful for decomposing the pure chemical from the multi-way fluorescence and mass spectroscopy data. The main difference between PARAFAC and PARAFAC2 models is that the strict multilinear assumption in the PARAFAC model is relaxed in PARAFAC2 model, meaning that the profiles for each slab in the multi-way data is not required to be the same in PARAFAC2 model if the cross products of the components keep the same [55].

In outline, mixture analysis holds significant importance in food analysis, and a range of chemometric tools are applied for this purpose. MCR-ALS is a powerful two-way method for mixture analysis, while PARAFAC and PARAFAC2 models are widely used in multi-way analysis of complex mixtures. These methods enable the extraction of chemically meaningful information from mixture data, aiding in the identification and characterization of individual components within complex food matrices. While certain challenges exist, strategies have been developed to address them and enhance the reliability and applicability of these chemometric tools in mixture analysis.

In this section, a general overview was provided regarding the various chemometric tools utilized for handling spectroscopy data. These tools encompass pre-processing techniques, exploratory analysis methods, variable selection approaches, regression models, classification algorithms, and mixture analysis methodologies. However, the specific methodologies and algorithms underlying each technique were not thoroughly explored, nor were the advantages or disadvantages of individual approaches discussed in detail.

## 3. Advanced Spectroscopy Techniques with Chemometrics in Food Analysis

Advanced spectroscopy techniques paired with chemometric tools are crucial in analyzing food by providing a fast, non-destructive, and efficient means of obtaining detailed information about food samples. This information can be used to improve the quality, safety, and authenticity of food products. Table 1 summarizes recent applications of advanced spectroscopic techniques and chemometric approaches for quantitative analysis in food whereas a summary of recent applications of advanced spectroscopic techniques linked to chemometric approaches for qualitative analysis in food can be found in Table 2. In this section, the focus was on discussing the most notable studies conducted on advanced spectroscopic techniques, including X-ray-based methods, hyperspectral and multispectral imaging, NMR, Raman, IR, UV, visible, fluorescence, and portable techniques. These studies were examined with respect to their applications, both qualitative and quantitative, and their overall utility in the field of food analysis.

### 3.1. X-ray-Fluorescence-Based Methods

Energy dispersive X-ray spectroscopy (EDXRF) is a technique commonly used for determining mineral content in food samples. Additionally, its association with the unsupervised and supervised data analysis tools demonstrated its efficiency to deal with the challenges of food analysis. The scope of this section is to discuss the application and usefulness of X-ray-based spectroscopic techniques in combination with chemometric tools for qualitative and quantitative analysis of various food samples.

For example, [57] EDXRF has been applied with PLS Regression for analyzing the micronutrient zinc in biofortified banana samples. This method showed good results mainly on low limits of detection (LOD) and quantification (LOQ). Another research work, conducted by Gamela et al. [56] used the same combination of EDXRF and PLS to determine not only zinc but also the contents of copper and strontium in cocoa bean samples. The study proved satisfactory results through the evaluation of developed PLS models in terms of the same criteria. Additionally, this study has been extended and proved the ability of EDXRF to be fused with Laser Induced Breakdown Spectroscopy (LIBS) to determine the micronutrient potassium in cocoa beans using the supervised technique of multivariate calibration. This fusion showed besides the satisfactory results an advantage to minimize the matrix effect induced by samples of cocoa beans.

The combination of Energy-Dispersive X-ray Fluorescence (EDXRF) and chemometric tools have been used for qualitative purposes too. Galvan et al. [113] carried out an analysis by EDXRF under two measurement conditions, to classify the geographical area of two food species and also according to the production mode by PLS-DA. These food species were tomato and sweet pepper samples Based on the good results of classification EDXRF was considered an excellent technique for authentication of plant-based food products based on the mineral elements K, Ca, Mn, and Fe. Another study for the same qualitative purpose [115] carried out by the association of X-ray Fluorescence (XRF) to (PCA) permitted to identify elements like Cl, K, Ca, Fe, Br, Cl, Rb and Sr which establish a clear fingerprint pattern of the tomato. Similarly, other work applied several chemometrics tools for the discrimination of Italian Extra Virgin Olive Oil (EVOO) geo-markers through the analysis of mineral constituents using EDXRF and associated with PCA and SIMCA [114]. Besides EDXRF and XRF, Total-Reflection X-ray Fluorescence was also employed for food screening [172]. For example, different wine samples from two different geographical regions of Croatia were discriminated against based on the analysis of thirteen metal contents through the association of TXRF with PCA, cluster analysis, and Linear Discriminant Analysis (LDA). Thanks mainly to PCA, elements such as K, Mn, Ba, and Ni were determined as the most relevant to characterize between different origins of wines [117]. TXRF has already been associated with both PCA to obtain the clustering of the bean seeds according to their geographical origin, then it was coupled to PLS-DA for classification purposes [116]. Specific studies that utilize EDXRF in combination with chemometric tools for qualitative and quantitative analysis of various food samples are highlighted. The end points emphasize EDXRF’s efficiency in determining mineral content, addressing challenges in food analysis, and its application in food authentication and geographical classification.

XRF-based methods are commonly used in food quality control and analysis due to their non-invasive and time-efficient nature. They can simultaneously detect and quantify trace elements and contaminants in food. However, they have limitations such as limited sensitivity, making them unsuitable for some applications, and being a surface analysis technique, they may not provide information on deeper layers of the sample. The presence of other compounds in the food matrix may also interfere with the analysis, necessitating calibration and standardization to minimize such effects.

### 3.2. Hyperspectral and Multispectral Imaging

In contrast to traditional spectroscopy, hyperspectral imaging affords continuous and high-resolution narrow-band spectral data linked to both physical and chemical sample composition [173]. With The HSI, an object’s spectral and spatial information can be retrieved simultaneously by integrating spectroscopic and imaging techniques. This technique has immense potential and has been reported in the detection of various food adulteration, especially when it is associated with chemometric approaches for quantitative purposes. The scope of the proposed paragraph is to discuss the application and advantages of hyperspectral and multispectral imaging combined with chemometric tools in detecting food adulteration, assessing food composition, monitoring food quality, and classifying different food products.

Various studies have been conducted recently on wheat flour to estimate its different contents [174]. Unlike conventional methods, HSI is a reagent-free, non-invasive [175]. One of the special HSI characteristics is to exhibit metabolic transformations, making it useful to assess food composition. A significant amount of recent work has been focused on the application of HSI to various food and agricultural products and animal products. For instance, hyperspectral imaging was used within 400–800 nm to develop a method for analyzing impurities of mites in wheat flour through the supervised chemometric tool of ANN [119]. Benzoyl peroxide, which can also be found as a bleaching agent in wheat [85], was investigated using shortwave infrared (SWIR) HSI and PLS regression [65]. The estimation of talcum content has also been done using hyperspectral imaging and the SNV-PLS model, which proved to estimate adequately the talcum content [64]. In terms of food analyzed by HSI, research by Al-Sarayreh et al. investigated the efficiency of hyperspectral imaging systems to detect meat adulteration, depending on its storage conditions. This analysis proved efficiently the advantage of CNN compared to SVM for this analysis purpose [120]. In addition to wheat and meat, HSI has also been applied to other food samples such as cheese. Priyashantha et al. developed and evaluated a predictive model based on coupling the NIR-HS imaging technique and PLS for determining the maturity state of cheese. The model was then applied on a pixel-wise basis, producing prediction images, and allowing for the determination of how and where the maturity spread in the cheese [58]. On the same food product, PLS and Monte Carlo Cross Validation (MCCV) were applied to HSI to detect the main wavelengths of fat and microbial transglutaminase (mTG), which is responsible for the color and yield of the cheese. Additionally, this study mentioned the possibility of using HSI to inspect the cheese remotely through its transparent foil [59]. Potato is another food sample that has recently started to be useful for monitoring its quality with HSI. Lu et al. assessed the impact of storage times on the evolution of solanine content in potatoes by using HSI in the spectral region of 500–1000 nm and support vector regression (SVR) and then allowed estimating the edibility of the potatoes [60]. Besides solanine content, the color is another indicator, that is also considered as a parameter to judge the quality of potatoes. Xiao et al. combined HSI in the region of 477–947 nm and used and compare two supervised tools: (PLS) and (LS-SVM) for predicting this last parameter [61]. The sweet potato has been subjected to HSI analysis. Tian et al. investigated how the moisture and total anthocyanin contents of potato samples under various drying conditions, by using HSI in the region of 400–1000 nm and PLS. The obtained results of this method showed a low prediction error and a high R^2^p [62]. The use of HSI has been shown to be useful in predicting specific parameters that characterize food products. Recently, Li et al. analyzed the quality of plum fruit based on color and soluble solid content using HSI and PLSR and showed how short-wave infrared (SWIR) hyperspectral imaging can predict soluble solid content, [63]. Sun et al. monitored the quality of melon through its indicators as sweetness and hardness by associating NIR hyperspectral imaging system to PLSR [66].

Besides the quantitative advantages of HSI that have been cited, HSI has also been widely used for qualitative purposes. HSI in the spectral domain has been applied to detect chilling injury in agri-food products, which could not be achieved without subjecting HSI to multivariate data analysis. For example, Cen et al. employed HSI in reflectance (500–675 nm) and transmittance (675–1000 nm) modes with supervised classification tools for the detection of chilling injury in cucumbers [124]. Tsouvaltzis et al. evaluated the chilling injury in eggplant fruit by coupling visible and Near-Infrared (NIR) HSI to classification tools such as PLS-DA, SVM, and KNN to classify eggplant fruit according to storage temperature [131]. Recently, Babellahi et al. demonstrated the convenience of HSI with PLS-DA to discriminate between cold-stored green peppers that can be impacted by chilling injury and fresh ones [123]. Another example applied to fruits; peaches might have chill damage during cold storage which Pan et al. associated HSI to Artificial Neural Network (ANN) to differentiate normal peaches from chill-damaged ones [121]. Sun et al. assessed the classification of peaches based on the chilling injury by PLS-DA, SVM, and ANN with Spectral Angle Mapper (SAM) which achieved the best classification performances [122]. Related to the application of HSI on peaches, Li et al. investigated and compared Long-Wavelength-Near-Infrared (LW-NIR) and Short-Wavelength-Near-Infrared (SW-NIR) hyperspectral imaging by associating them with PCA and the approach of watershed segmentation for discriminating bruised from healthy peaches [118]. This study clearly proved the advantage of SW-NIR in detecting early bruises in peaches. Moreover, bruises have also been identified in blueberries samples by SWIR hyperspectral image and the developed models based on two approaches: considering Least Squares-Support Vector Machine (LS-SVM) with full spectra and optimum selected wavelengths by (CARS) (CARS-LS-SVM model) [126]. There are many factors that can lead to bruising food items. For example, Hyperspectral imaging technology applied in the region 400–1000 nm, was used with PLS-DA to classify bruised tomatoes that were caused by falling damage, detection times, falling heights or fruit sizes [127]. Besides fruits and vegetables, HSI with chemometric tools has shown its convenience in the qualitative analysis of wheat. Zhao et al. developed an approach based on a hybrid CNN model with hyperspectral imaging technology to classify different varieties of wheat seed [129,176]. In addition to wheat seeds, maize seeds were classified through the association of HSI with the chemometric approach of Radial Basis Function Neural Network (RBFNN) [130]. Soares et al. also presented a new strategy for fast and non-destructive classification of cotton, based mainly on coupling NIR-HSI images to PLS-DA. The results of this method showed good accuracy in the classification of test samples, with correct classification rates [125]. The endpoints of this section are to emphasize the potential of hyperspectral and multispectral imaging in providing continuous and high-resolution spectral data linked to physical and chemical composition, their non-invasive and reagent-free nature, and their ability to analyze various food samples.

Hyperspectral and multispectral imaging are valuable tools for food quality control and analysis, with advantages and limitations to consider. These imaging techniques offer non-destructive analysis and high spatial resolution for detailed surface analysis, simultaneous detection of multiple analytes, and real-time analysis for efficient quality control. However, hyperspectral, and multispectral imaging equipment can be expensive, have limited penetration for internal composition analysis, may lack sensitivity for detecting low analyte levels, and require complex image processing and specialized expertise for data analysis.

### 3.3. Infrared Spectroscopy

Infrared including (NIR) and (MIR) are ones of the conventional spectroscopy that have been usually used with many multivariate data analysis tools in food quantitative analysis. The scope of the proposed paragraph is to discuss the application and benefits of infrared spectroscopy with chemometrics, in quantitative and qualitative analysis of food components, such as carbohydrates, proteins, fats, and moisture content, as well as the determination of functional groups, carbon, and nitrogen.

MIR is responsible mainly for detecting functional groups as well as carbon and nitrogen, whereas NIR is used for determining carbohydrates, proteins, fats, and moisture content in various foods [177]. However, the method is not sensitive enough for samples containing just small amounts of target components. The fundamental vibrations occur when absorbed in the NIR [178]. Wang et al. combined NIR PLSR to estimate the content of potato flour in steamed bread [67]. Recently, the same association of NIR with PLS regression was applied to wheat flour samples to estimate the quantity of low-content talcum. In this study, several chemometrics were applied with PLS together to select the effective feature as genetic algorithm and elastic net, thus improving the capacity of the PLS model [68]. In addition to talcum, zearalenone might have an impact on the quality and safety of wheat grains. Recently, a study was carried out to determine zearalenone in wheat by NIR spectroscopy and (SVM) model. The results were significantly improved after the application of a variable selection approach called least absolute shrinkage and selection operator (LASSO) to extract useful spectral regions of NIR. In contrast to the contents that can have an impact on wheat, the determination of valuable contents has been featured in many recent research works [73]. Kamboj et al. predicted quality parameters mainly protein and carbohydrate of wheat content that has been stored at different temperatures using NIR Spectroscopy (NIRS) with PLS, MLR, and SVM [70]. Additionally, the fatty acid value is also considered an important indicator of the quality of wheat flour, particularly during storage. Therefore, Jiang et al. demonstrated the feasibility of using portable NIR spectroscopy in conjunction with appropriate chemometric methods to achieve quantitative determination of fatty acid values in wheat flour during storage. Jiang et al. used a method called variable combination population analysis (VCPA) in addition to PLS to improve NIR spectral characteristic wavelengths [72]. In addition to the chemometric tools that were cited and used for quantitative purposes, MCR-ALS is one of the chemometric tools that is combined with FT-NIR spectroscopy to estimate certain characteristics of food samples. For instance, an assessment of the combination of multivariate tools, including PLS regression and MCR-ALS, was used to predict antioxidant activity from clove and pomegranate extracts. The results showed that MCR-ALS with FT-NIR stood out among PLS with high R^2^ and low RMSEP [74]. Another application of PLS and MCR associated with FT-NIR was used successfully to estimate peanut oil adulterants, [75]. Castro et al. also proved the efficiency of coupling FT-NIR with MCR-ALS for the quantitative purpose of four adulterants at low levels in a complex mixture of saffron, including onion, calendula, pomegranate, and turmeric [76]. In terms of determining adulterants in saffron, PLS-R was applied to FT-NIR data of saffron to estimate lotus stamens and corn stigmas. This study proved the efficiency of combining PLS with the variable selection approach of competitive adaptive reweighted sampling (CARS) showing good results [77]. Additionally, crocin I and II were analyzed using near-infrared spectroscopy and chemometrics. Crocin I and II are considered the most important indicators of the quality and commercial value of saffron [179]. Le et al. used FT-NIR and PLS to determine these two contents in saffron with low RMSECV [78].

Many studies have shown how MIR and NIR spectroscopy are efficient for the qualitative analysis of different food varieties comprised for example identification, classification, and authentication, based on, for example, country of origin. For instance, Liang et al. used NIR spectroscopy appropriately for the detection of zebra chip disease using Canonical Discriminant Analysis with a low classification error rate [71]. Discriminative analysis was applied to durum wheat to determine if they were contaminated by ochratoxin by combining FT-IR and FT-NIR with PLS-DA and PCA-LDA. In this study, FT-IR and FT-NIR were convenient spectroscopic techniques for discrimination purposes [133]. PLS-DA was compared to other classification tools such as HCA, SVM, and ANN to identify and classify Panax notoginseng with its adulterants. The classification purpose of this work was achieved by both PLS-DA and SVM with 100% classification accuracy [79]. PLS-DA showed its efficiency in detecting the freshness of rice based on storage time using FT-NIR with an accuracy of 96%, whereas the application of KNN achieved an accuracy of 100% [136]. In relation to the analysis of rice by NIR, L.-H. Xie et al. led a discrimination of two kinds of rice, waxy, which contains very low apparent amylose content, and non-waxy rice. The developed PLS-DA model allowed the recognition of these two types of rice with 100% accuracy [139]. Detecting fake eggs from authentic ones is another example that proves the efficiency of FT-IR and chemometrics in this field of food analysis. Joshi et al. showed how PLS-DA and SVM achieved a good classification of 100% [81]. The authenticity of the native was subjected to FT-NIR analysis by Chen et al. who proved the efficiency of using Data-Driven Class Modeling (DD-SIMCA) as an alternative tool for this classification [134]. The end points of the paragraph are to emphasize the effectiveness of infrared spectroscopy in estimating the content of target components in food samples, such as potato flour, talcum, zearalenone, protein, carbohydrate, fatty acid values, and antioxidant activity. The paragraph also mentioned the successful application of chemometric approaches in enhancing the accuracy and reliability of quantitative analysis using NIR and MIR spectroscopy.

Overall, infrared spectroscopy is a powerful and versatile tool for food quality control and analysis. However, limitations such as limited penetration, sample homogeneity requirements, calibration requirements, complexity of data analysis, and interference from other components should be taken into consideration when using this technique.

### 3.4. Raman Spectroscopy

Raman spectroscopy is a vibration spectroscopy technique that is based on monochromatic light diffusion. It involves the excitation of a sample by collisions with photons, which causes the sample to reach an unstable state of virtual energy. The scope of the proposed paragraph is to discuss the application of Raman spectroscopy in food analysis, both qualitatively and quantitatively.

Raman spectroscopy was carried out for the determination of fat content in various food samples such as milk and meat. Heterogeneous foods have recently been detected chemically with Raman microscopy [180]. This combination is used qualitatively and quantitatively to evaluate food value. Many organic components are detected and identified based on the absorption curves. Microscopic food species can be analyzed too. Raman microscopy has been carried out to determine the main composition of wheat and to detect protein content changes during milling [181,182]. Raman spectroscopy detects changes in protein secondary structure, conformational changes in lipid-binding proteins.

Based on the Raman spectrum, it is possible to estimate the relative concentration of food contents. For instance, a recent study aimed to determine starch using Raman spectroscopy and a linear regression model for a specific band, and PLS regression for a specific spectral region [183], which confirmed the efficiency of association of FT-Raman to PLS for the estimation of gluten content in flour [184]. Carotenoids have been determined in tomatoes by Raman spectroscopy and PLS regression and proved low prediction error [88]. The main characteristic of Raman spectroscopy is that it can directly measure aqueous solutions because of the low effect of water, and even the sample preparation of liquids for Raman analysis is simple, which can be considered an advantage to estimating the contents in food liquids such as milk [185]. Whey is one of the contents that has been quantified accurately in the milk [89]. In addition to whey, macronutrients such as fat, lactose, and protein have been successfully quantified in commercial yoghurt samples using FT-Raman spectroscopy and PLS models [90]. In a recent study, milk adulterants such as sodium bicarbonate, maltodextrin, and whey were also analyzed using Raman spectroscopy and the PLS chemometric tool, with a low detection limit [91]. A handheld Raman spectrometer has also been applied to quantify lard in adulterated butter, another milk derivative, through PLS [186]. Richardson et al. demonstrated how Raman spectroscopy is able with PLS to detect coconut water adulteration [112]. In a recent study, various variable selection approaches were tested on surface-enhanced Raman scattering spectra of rice, used with PLS for quantifying the target residue analyte of chlorpyrifos [109].

As it has shown its relevance for quantitation, Raman spectroscopy has proven its efficiency with chemometric tools in many recent studies. For example, Robert et al. built a classification model using SVM and PLS-DA to discriminate lamb meat from beef meat despite the similar chemical composition of these two species [147]. Hai Chao et al. classified duck meat according to the residues of testosterone propionate and testosterone nandrolone using Raman Spectroscopy and Support Vector Classification (SVC) which shows a classification rate of 100% for the test set [86]. Other residues that have an impact on duck meat and have been subjected to analysis by means of Raman spectroscopy and chemometrics are sulfonamides, comprised of sulfadimidine and sulphapyridine [187]. A recent research work used a support vector classification on Raman Spectroscopic data to classify duck meat into four groups, which are as follows: samples free of residues, samples containing one of the two residues, and samples containing both residues [87]. Another mode of Raman spectroscopy called Spatially Offset Raman Spectroscopy, which allows to measure the chemical compounds under the surface of meat tissues [188]. Besides that, a study has also proven that the use of Raman spectroscopy in combination with the SVM method can discriminate rice samples according to their regions with high-rate accuracy [148]. Raman was applied to differentiate four categories of milk species of cow, buffalo, goat, and human. Thus, Principal Component Analysis (PCA) besides Random Forest (RF) was applied on Raman data to highlight and characterize the Raman spectra of different milk samples with high accuracy of 93.7% [144]. In addition to benchtop Raman, Handheld Raman spectroscopic devices have shown their efficiency using SIMCA to classify milk samples from adulterated ones [189]. In addition to milk and its derivative samples, PLS-DA was employed with Raman to accurately classify a milk derivative of cheese whether it was adulterated by starch or not [93]. Related to handheld Raman, Aykas, et al. succeeded in seeking to characterize commercial honey by combining handheld Raman equipment and SIMCA [190]. A recent research work monitors according to a new method the adulteration in cassava starch, by means of Raman spectroscopy and supervised tool One-Class Modelling (OC-SVM) which proved its higher accuracy compared to the SIMCA, allowing for the discrimination of samples [191]. Discriminant analysis by PLS-DA of coffee genotypes by Raman spectroscopy based on two main contents, kahweol and fatty acids, has shown how Raman with chemometrics was more effective compared to sensorial analysis [192]. Sha et al. combined Raman with PCA, HCA, and SVM for feature extraction to improve the efficiency of identification of rice varieties [176]. For oil samples, Jiménez-Carvelo et al. used chemometrics for the classification and characterization of pure olive oil from adulterated using Raman spectroscopy in addition to NIR by employing classification models. While PCA was used to reduce the features, other supervised techniques were applied to for the discrimination goal [193]. Raman analysis was also employed to discriminate waste cooking oil from edible vegetable oil. Thanks to PCA, signals at 869, 969, 1302, and 1080 cm^−1^ were found to be the most important features to differentiate between these two types of oils. In addition, PCA demonstrated its ability to separate adulterated from pure oils when the adulteration proportions reached 10% and 20% [194]. The endpoints of this part include the successful application of Raman spectroscopy in estimating the content of specific food components and the detection and classification of various residues and adulterants in food samples.

Finally, Raman spectroscopy is a valuable method for analyzing and controlling the quality of food, offering several advantages such as non-destructiveness, molecular specificity, sensitivity, minimal sample preparation, and high spatial resolution. However, when using this technique, some limitations must be considered, including its limited penetration depth, susceptibility to fluorescence interference, equipment cost, complexity of data analysis, and sensitivity to water.

### 3.5. NMR Analysis

Nuclear magnetic resonance (NMR) is a spectroscopic technique used to determine the molecular structure and physical properties of substances, and efficiently used to ensure the quality of different varieties of food samples [195]. The scope of the proposed paragraph is to discuss the application of NMR spectroscopy in food analysis, both qualitatively and quantitatively. It focuses on the use of NMR spectroscopy and chemometric tools for food identification, discrimination, and characterization purposes.

For example, the combination of low and high-field NMR and chemometrics, including PLS-R and SVR, has proved its ability to accurately estimate essential quality parameters of edible oils, especially to detect potential adulteration. The results summarized in statistical parameters indicate that all developed models, whether of PLS-R or SVR on the three different fields of NMR, were similar. In addition to oil applications, Haddad et al. have carried out a quantitative analysis of fatty acids based on ^1^H-NMR variables as predictors and relative mass percentages of fatty acids as targets, including caproic, caprylic, capric, oleic, palmitic, and margaric [110]. Fatty acids have been accurately estimated in hen egg samples by ^1^H-NMR and PLS regression [108]. Proton nuclear magnetic resonance (^1^H-NMR) associated with chemometrics were combined to investigate the camellia oil adulterants with other vegetable oils [170]. In addition to ^1^H-NMR, ^1^H TD-NMR was combined efficiently with PLS regression to detect the percentage of adulterants such as water and whey in milk products varied from 5% to 50% through milk package and without sample preparation [105]. Besides PLS regression, Sun et al. successfully set up a model to detect moisture content through the association of low-field NMR and ANN with a low RMSE [107].

As previously shown, NMR spectroscopy supported by multivariate data analysis tools has been applied for various qualitative purposes in different foods. For example, Milani et al. successfully explored the versatility of 1H NMR with pattern recognition of PCA and SIMCA for identification and discrimination of pure Brazilian coffee from adulterated ones by corn, barley, or even coffee husks. The built SIMCA model ensured its high classification accuracy [168]. In relation to these quality analysis of coffee, 1H NMR data of roasted coffee samples were analyzed qualitatively by OPLS-DA to characterize organic roasted coffee from conventional coffee. The orthogonal signal correction (OSC) allowed for the extraction of the main features of each coffee category and thus improved the PLS-DA model discrimination. While fatty acids, β-(1-3)-d-galactopyranose, quinic acid, and its cyclic ester were the major metabolites characterizing organic roasted coffee, conventional coffee was characterized mainly by trigonelline and chlorogenic acid isomers [163]. The OSC filter was used with PCA (OSC-PCA) and applied to HR MAS 1H NMR data of cocoa beans to discriminate them based on their origin, whether they were American or African, based on the fatty acids, acetate, and saccharides components [167]. In another research work, both 1H NMR and 13C NMR were employed to analyze refined edible oils from different sources. By applying PCA on the 1H NMR or on 13C NMR, it was possible to identify and characterize these plants based on their fatty acids [171]. Amino acids were analyzed by NMR and explored by chemometric tools in fruits, since they are considered essential metabolites in cell function and enable distinction between plants of the same fruit. For example, Botoran et al. identified ten kinds of amino acids that allowed for the observation of differences and distinctions of different varieties of juice using PCA and LDA, which accurately classified juices from different plant sources [162]. In the honey adulteration problem, Rachineni et.al analyzed successfully honey by associating 1H NMR with supervised machine learning (neural network) for the characterization purpose of authentic honey from the adulterated whether by sugar, brown rice syrup or jaggery syrup [169]. The endpoints of the paragraph include the successful application of NMR spectroscopy combined with chemometrics, and machine learning, in accurately estimating and detecting various quality parameters and adulterants in food samples.

Overall, NMR spectroscopy is a valuable tool for food quality control and analysis, offering numerous advantages such as non-destructiveness, molecular specificity, sensitivity, and versatility. Additionally, it allows for quantitative analysis, making it particularly useful for determining the concentration of specific compounds in food products. However, the technique also has limitations that should be considered, including equipment cost, limited penetration, sample preparation requirements, and sensitivity to sample properties.

### 3.6. UV-Visible

UV-visible spectroscopy is known as one of the most sensitive techniques for determining less concentrated contents in food samples. Its association with multivariate tools offers an added advantage for such quantitative analysis. This technique uses electromagnetic radiation between 200 and 800 nm and detects two different aspects: color and fat oxidation [104]. The scope of the proposed paragraph is to discuss the application of UV-visible spectroscopy in food analysis, particularly for quantitative and qualitative purposes.

In addition to other analytical techniques, a recent research work for the same food product employed UV-Vis with PLS regression to accurately determine squalene in Extra virgin olive oils (EVOO) [100]. Wu et al. integrated empirical mode decomposition with SVR (EMD-SVR) to evaluate the quality of edible blend oils samples, concluding that EMD-SVR was more accurate for the quantitative analysis of ternary edible blend oil [101]. Zhang et al. developed models by coupling UV-Vis to Partial least squares regression (PLS) and principal component regression (PCR) for the quantitation of acid value in various oils. The PLS models performed well compared to PCR models [102]. In addition to different oil analyses, UV-Vis spectroscopy was proved to be more efficient as a method associated with PLS instead of univariate tools for the quantitation of grape-must caramel in Balsamic vinegars of different varieties of wine vinegars [103].

The UV-Vis spectroscopy has been combined with multivariate techniques for various qualitative purposes in food analysis. For instance, UV-Vis combined with MCR-ALS is a suitable tool to pursue the autoxidation of edible oils and to monitor the quality of extra virgin olive oil (EVOO) in different packaging systems [157]. In addition to olive oil samples, multivariate discrimination tools using UV-Vis spectroscopy, such as PLS-DA and SVM, have been used to distinguish between two specific mint species, such as spearmint and peppermint, while SIMCA has been used to detect outlier samples other than the two species [161]. Similarly, coffee has been analyzed by UV-Vis spectroscopy and SIMCA to accurately classify and discriminate between Peaberry and normal coffee [158]. In addition to SIMCA and PLS-DA, artificial neural networks (ANN) have been applied to UV-Vis spectroscopy to discriminate between vinegars produced from different raw materials, showing the discrimination efficiency compared to PLS-DA [159]. Another study used UV-Vis spectroscopy and ANN to discriminate between vinegars adulterated with spirit vinegar or acetic acid [160]. The end points of the paragraph include the successful application of UV-visible spectroscopy combined with multivariate techniques for food analysis adulteration, improved discrimination, and classification purposes.

Generally, UV-Visible spectroscopy is a valuable tool for food quality control and analysis, with several advantages such as simplicity, non-destructiveness, versatility, and cost-effectiveness. However, it has limitations in sensitivity, interference, and surface analysis that should be considered while using this technique.

### 3.7. Fluorescence Spectroscopy

Fluorescence spectroscopy is a technique that focuses mainly on the molecular level. It refers to the process in which a specific wavelength of light is irradiated in a solution, and the fluorescent substance in the solution absorbs the released energy. The scope of the proposed paragraph is to discuss the application of fluorescence spectroscopy in food analysis, both for quantitative and qualitative purposes. It highlights the molecular-level focus of fluorescence spectroscopy and its ability to detect and analyze various elements in food samples.

In recent years, fluorescence spectroscopy has been applied for the analysis of various elements of food. For example, a recent research study exhibited the application of front-face fluorescence mode spectroscopy and supervised PLS to estimate cow milk adulteration with other milk kind [196]. Another study used excitation-emission matrix (EEM) fluorescence spectroscopy and second-order calibration ways, like (PARAFAC) and (U-PLS), to detect and estimate the content of melamine in milk [98]. Additionally, fluorescence spectroscopy has been used to detect and quantify adulteration in olive oils [156]. Three-dimensional fluorescence spectra were subjected to analyze the same analysis purpose using the supervised approach of GA-SVR [99].

Many studies have shown the potential of fluorescence spectroscopy combined with multivariate data analysis tools for the qualitative analysis of various food samples. For example, Yuan et al. [155] conducted a comparative study using excitation-emission matrix fluorescence, FTIR, and vis-NIR on different types of vegetable oils for discrimination purposes using advanced chemometric tools (PCA, multiway-PCA, PLS-DA, and unfold-PLS-DA). The study found that FTIR and Vis-NIR, were more suitable compared to EEM for the identification of vegetable oil species. This is because most chemical components in vegetable oil produce FTIR and NIR absorption, while only a small number of fluorophores produce fluorescence [155]. Another study proved the same classification results of these techniques for detecting olive oil adulteration. This highlights the importance of combining analytical techniques with the appropriate chemometric tool [154]. Fluorescence spectroscopy has been combined with chemometrics to distinguish pure Aroeira honey from adulterated. The advanced chemometric methods (PARAFAC, PLS-DA, unfolded PLS-DA (UPLS-DA), and N-way PLS-DA (NPLS-DA)) were used to decompose the spectral data and build classification models. This qualitative analysis has proven the convenience of fluorescence spectroscopy with UPLS-DA for this kind of honey analysis [151]. It can be noted from previous research that multi-way chemometric techniques are often applied conveniently to EEMs data, whether on edible oils, honey, or beverages, as demonstrated by Fang et al. for the classification of Chinese lager beers made by different manufacturers [152]. The end points of the paragraph include the successful application of fluorescence spectroscopy combined with multivariate data analysis tools for quantitative and qualitative analysis of various food samples.

Fluorescence spectroscopy is a powerful tool for food quality control and analysis, offering advantages such as high sensitivity, specificity, non-destructiveness, and rapid analysis. However, there are certain limitations that should be considered when using this technique, including the complexity of sample preparation, potential interference from other compounds, limited penetration depth, and high instrumentation costs.

### 3.8. Fusion of Spectroscopic Techniques

In recent years, the strategy of data fusion combined with multivariate statistical analysis, that has been widely used to ensure the safety of food and to extract more information for both qualitative and quantitative purposes. The scope of the proposed paragraph is to discuss the application of data fusion combined with multivariate statistical analysis in food analysis for both qualitative and quantitative purposes. It highlights the use of various spectroscopic techniques such as UV-VIS, NIR, Raman, FT-IR, FT-Raman, and MID, and their fusion with multivariate statistical models for food analysis.

A recent research work developed PLS and ANN models for the quantification of adulteration in honey, using data fusion of non-pre-processed UV-VIS and NIR spectra [96]. Vis-NIR and Raman have also been merged and applied to predict the storage time of infant formula between 0–12 months [95]. UV-Vis-NIR was combined with PLS to accurately quantify cholesterol in egg yolk, whether in the shell or in pasteurized form [94]. Additionally, the combination of PLS regression with the data fusion of FT-IR with Raman spectroscopy allowed the determination of peroxide values and acid values in oils [197]. A study was elaborated to test merging mid-infrared (MIR) with Raman spectroscopy for the fructose syrup determination in honey samples. The PLS model was used to estimate the adulterant, and the results were improved after the data fusion compared to the results obtained by each of the two spectroscopic techniques [198]. The same conclusion was achieved by a recent research work that evaluated the data fusion of NIR and MIR, combined with the sequential orthogonalized partial least square regression (SOPLS), to estimate different quality traits of tubers and root flours. These traits included different chemical compounds including for example amylose and protein [69].

The efficiency of data fusion methodology for qualitative purposes has been proved by Yao et al., who established a method based mainly on Fourier transform infrared (FT-IR) and ultraviolet (UV) spectroscopies associated with data fusion to distinguish different regions of mushroom samples [150]. A synergistic strategy of FT-Raman and NIR for the classification of two classes of hazelnut: unadulterated and adulterated with almonds using SIMCA was also demonstrated. The obtained results proved that merging the two techniques can be more effective than using each technique alone, based on sensitivity and specificity [142]. NIR and MID were also used with the SVM model to discriminate natural honey from syrup-adulterated one. In this case study, Huang et al. showed two levels of data fusion. A low level, in which redundant and irrelevant variables were introduced, and an intermediate level, where PCA was applied to extract the feature variables. The results acquired from this study had a significant increase in SVM model parameters of accuracy, precision, and sensitivity using the intermediate-level data fusion [132]. The endpoints of this section include the successful application of data fusion methodologies with chemometrics for quantification of adulteration, for estimating different chemicals, as well as for qualitative purposes and classifying unadulterated and adulterated food samples. However, there are limitations to consider when using data fusion methodologies in food analysis. The success of data fusion relies on the compatibility and complementarity of the combined techniques and the availability of appropriate statistical models. Proper calibration and validation procedures are necessary to ensure the reliability and robustness of the fused data. Furthermore, data fusion may introduce additional complexity and computational requirements, requiring careful data preprocessing and analysis. It is also important to consider the specific requirements and limitations of each spectroscopic technique and statistical model being used for data fusion.

The fusion of spectroscopic techniques provides an advanced tool for food quality control and analysis, offering advantages such as enhanced accuracy, complementary information, improved sensitivity, and non-destructiveness. However, it should be noted that this technique has some limitations, such as complexity, high equipment cost, sample preparation requirements, and limited penetration depth. These factors should be considered before implementing this technique for food analysis.

### 3.9. Portable Spectroscopic Techniques

The food industry is constantly seeking faster, more accurate ways to assess the safety and quality of their products while also detecting possible adulteration. Portable spectroscopic equipment, such as Raman, NIR and HSI among other techniques, have become increasingly popular due to their portability, accuracy, and ability to control food products [199]. The scope of the proposed paragraph is to discuss the application of portable spectroscopy techniques in the food industry for assessing food safety and quality, detecting adulteration, and enabling in-process monitoring. It highlights the advantages of portable spectroscopy equipment, such as Raman, NIR, and HSI, including their portability, accuracy, low sample preparation requirements, and cost-effectiveness.

Portable spectroscopies equipment in general requires less sample preparation and less hazardous consumption than traditional laboratory-based processes, granting fast results on food quality and safety. Furthermore, these technologies provide the food industry the low cost-effective analysis and product safety [200,201]. Portable spectroscopy techniques are highly effective in detecting food fraud and contaminants, such as pesticides, heavy metals, and pathogens. Previous developments in portable fluorescence and Raman spectroscopy have enabled water detection in milk and honey products with less expensive syrups, respectively [202,203]. Moreover, they are involved to analyze food quality attributes, for instance, mid-infrared spectroscopy used for fatty acid profile and fat content in lamb meat [202], and the amount of fat in meat and its degree of tenderness [204]. The NIR-HSI as a portable technique coupled with chemometric tools was showed excellent application in different food products [205,206].

Portable spectroscopy techniques were demonstrated to be a valuable solution for food manufacturers and processors, as they can be used for in-process monitoring of food quality and safety. The technology can also be used for post-harvest processing, such as the detection of mold and fungal infections in food products [207]. In addition, they have demonstrated to be versatile in identifying various food tampering and contaminants, including pesticides, heavy metals, and biological contaminants. They are also beneficial with chemometrics for quality control testing and authentication for rapid food chain analysis aimed at a perfect digital traceability system [201]. For a deep understanding, a recent review article provides an overview of how miniaturized NIR spectroscopy can be applied to address a range of issues in food-related settings [208]. They provide a comprehensive summary of the latest research trends, highlighting key factors driving the development of the micro-NIR analytical framework for modern food analysis, quality control, and safety risk monitoring. Emphasis is placed on the significance of combining complementary tools with the NIR analytical method, which enhances its precision, dependability, and versatility for food applicability. The endpoints of this section include the successful use of portable spectroscopy techniques and the use of chemometric tools for detecting food fraud and contaminants, analyzing food quality attributes, monitoring in-process food quality, safety, and rapid food chain analysis.

Finally, to highlight portable spectroscopy tools have several advantages in food applications, including non-destructive sample analysis, rapid analysis where timely decisions are needed, in situ analysis which is particularly useful in food-based applications. However, there are also some limitations to the use of portable spectroscopy techniques in food applications, such as limited accuracy particularly for complex samples, limited range of wavelengths which may not be suitable for all applications, regular calibration requirements which can be time-consuming, sensitivity to environmental conditions such as temperature and humidity, and often more cost-effective than traditional laboratory-based techniques [201]. These limitations should be taken into account when implementing portable spectroscopy in food analysis and quality control processes.

## 4. Importance of Integrating Advanced Spectroscopy Techniques in Food Analysis

**Figure 3:**
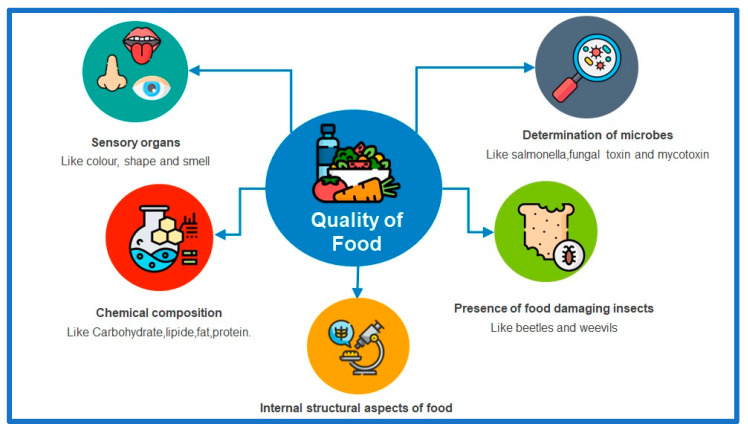
Factors that affect the quality of food.

The use of advanced spectroscopy techniques in food analysis is crucial for several reasons. The scope of the proposed paragraph is to discuss the significance of advanced spectroscopy techniques in food analysis, including their non-destructive nature, ability to provide detailed information about food composition, and the use of chemometric tools for data analysis. Firstly, techniques such as NIR, Raman, FTIR, and UV-Vis offer a non-destructive and non-intrusive way of analyzing food samples, making them ideal for large-scale analysis in the food industry without affecting the quality or safety of the samples. Secondly, they provide detailed information about the chemical composition and structure of food, which can be used to classify and identify different food types, detect contamination, and monitor food quality changes. Lastly, the implementation of advanced chemometric tools enables effective analysis of complex spectral data for food quality assessment. The food industry faces a significant challenge in ensuring consistent quality and overall food safety information throughout the entire supply chain, from production to distribution, to meet consumer demands and expand the market. To address this challenge, advanced non-invasive technologies are increasingly being used to monitor the nutritional and hygienic properties of raw and end food products. The initial perception of a food’s quality is often based on its texture, while the nanostructures in food play a role in determining its color, shape, and sensory appeal (Figure 3).

Spectroscopy techniques can help to determine the presence and quantity of key components like flavonoids and antioxidants in food, which play a vital role in assessing its quality. The effectiveness of using anthocyanin profile, color image analysis, and NIR-HSI to differentiate between different grape varieties, with the aid of Stepwise Linear Discriminant Analysis (SLDA) that was created for each dataset to differentiate grapes based on their variety.

Spectroscopic methods provide a valuable means to assess the quality of food by determining the presence and quantity of important components such as flavonoids and antioxidants. The combination of anthocyanin profiling, color image analysis, and near-infrared hyperspectral imaging (NIR-HSI), along with the aid of dataset-specific Stepwise Linear Discriminant Analysis (SLDA), proves effective in distinguishing between different grape varieties based on their specific characteristics. In addition, it can be concluded that NIR spectroscopy holds substantial promise for the non-destructive determination of total phenolics and flavonoids [209]. The content of flavonoids in food can be influenced by various factors, including seasonality, food maturity, and preparation methods employed. In this sense, the spectroscopy methods such as Visible/NIR were used to determine the levels of flavonoids in food [210]. These techniques are employed by various industries including the milk, meat, coffee, and wine sectors to analyze the composition of food [211]. Assessing the quality of food based solely on sensory evaluation may not suffice, particularly for intricate evaluations. Advanced spectroscopy equipment with chemometrics is highly recommended in such scenarios as it offers advantages that are not attainable through manual inspection alone.

Traditional methods of food quality detection can be cumbersome, repetitive, destructive, and take up a lot of time. Non-destructive methods, on the other hand, offer a more efficient way of gaining both quantitative and qualitative data without destroying the sample. The recent developments in non-destructive food quality assessment techniques include imaging, spectroscopy, and cutting-edge approaches such as electronic nose, electronic tongue, dielectric, and acoustic methods [212]. For instance, conventional methods of analysis and detection, for instance, thin-layer chromatography and high-performance liquid chromatography (HPLC) are yet largely used in the basic food industry to detect food quality. Nevertheless, these procedures are damaging, laborious, and time-consuming. Consequently, spectroscopic techniques were demonstrated their use and importance for food quality control including visible/infrared (VIS/IR) [212,213], Raman spectroscopy [214], NMR [215], and HSI [216].

In contrast, the field of food authentication poses a major challenge that can be addressed using advanced spectroscopic tools. Adulteration in food is often accomplished by substituting ingredients with cheaper options that are not easily distinguishable by either consumers or conventional analytical techniques [217]. To confirm the geographical origin, storage conditions, and processing methods listed on food labels, it is necessary to analyze specific components in samples. Hence, the development of trustworthy and effective analytical methods is vital for creating new policies, programs, and techniques to verify food authentication.

The scope of the proposed paragraph is to emphasize the importance of advanced spectroscopy techniques in food analysis, including their non-destructive nature, ability to provide detailed chemical information, and the role of chemometric tools in analyzing complex spectral data. The endpoints are to highlight the use of spectroscopy techniques for assessing food quality, detecting adulteration, and verifying food authentication. However, it should be noted that the paragraph does not delve into the specific methodologies or algorithms associated with spectroscopy techniques and chemometric data analysis. However, there are limitations to consider when using spectroscopy techniques in food analysis. These limitations include the influence of various factors on the content of flavonoids in food, the need for dataset-specific analysis methods, and the challenges associated with distinguishing food adulteration using conventional analytical techniques.

In summary, the integration of different spectroscopy techniques either separately or in a combination or fusion can also enhance the overall performance of food analysis. This can be achieved by using chemometric data analysis approaches.

## 5. Food Application and Aspects

Spectroscopic methods involve the use of electromagnetic radiation through absorption, transmission, and emission, and are based on the wavelength of the radiation. Unlike traditional techniques, spectroscopic methods have become essential tools for determining food properties through real-time monitoring and non-invasive techniques. Recently, advanced spectroscopic techniques combined with chemometric approaches have been developed and applied in various food applications and areas such as sensory analysis, adulteration detection, chemical analysis, mycotoxin detection, parasitic infection detection, internal physiological analysis, and others.

### 5.1. Sensory Attributes

The sensory characteristics of basic foods, such as structure, color, toughness, texture, and outside defects, are crucial elements of food quality. The color of a food is generated by the reflection of distinct wavelengths in the visible light section. The structure of a product, which is often determined by its size, weight, or volume, can influence consumer preferences and final consumption. Hardness is a primary indicator of food property, indicating the texture and moisture content of food. External defects, which occur during or after harvest, are another common sensory attribute that greatly impacts food quality. Consequently, precise, and timely forecasting of these sensory characteristics is a primary concern for the farming and food industry. The scope of the proposed paragraph is to discuss the importance of sensory characteristics in food quality and the role of spectroscopy techniques combined with chemometrics in evaluating these characteristics. It highlights the significance of texture, color, hardness, and external defects in determining food quality and consumer preferences.

The sensory characteristics of texture and color were established using VIS and NIR spectroscopy. The VIS/NIR spectroscopy was shown to be a reliable method for evaluating the quality of dry beans during the canning process [218]. A discriminatory linear model was applied to classify the canned beans into two categories: “acceptable” and “unacceptable”. The model achieved an ordinary classification accuracy of 72.60%. As sensors and instruments improve, it is expected that VIS/NIR spectroscopy will meet the necessary requirements for its use. The evaluation of basic foods using HSI systems mainly focuses on color, hardness, and external defects. In a recent study [219], the color of fresh soybeans was determined using an active contour model to segment the spectral images. This HSI technique was found to be more effective in acquiring the mean reflectance and entropy parameters of the image. A PLSR model was established to detect the color of processed soybeans with a good R^2^ of 0.74 and used HSI in the range of 400–1000 nm to discriminate rice samples based on color and shape. Five shape features (minor axis length, major axis length, perimeter, length-width ratio, and eccentricity) and one-color feature (degree of chalkiness) were utilized as inputs for a back propagation neural network (BPNN) model, resulting in a high classification accuracy of 94.45%. The performance of PLSR and PCR in evaluating the hardness of wheat samples using HSI in the wavelength range of 960–1700 nm and found that PLSR outperformed PCR. Additionally, hyperspectral imaging was also significantly used to determine the surface defects of potatoes [220]. The effectiveness of the proposed method in identifying kernels where the germination process has begun was demonstrated through experiments involving three wheat cultivars. The results achieved 100% accuracy for the samples utilized in this study [221]. The surface defects of potatoes were also determined using the HSI technique. Moreover, the results were combined with the SVM classification tool to achieve a high-accuracy performance in determining the surface defects of potatoes [222]. The endpoints of the paragraph include the effectiveness of spectroscopy techniques, such as VIS/NIR and HSI, in determining the color, hardness, and external defects of food samples. It also highlights the successful application of discriminatory models and neural networks in classifying and predicting sensory characteristics based on spectroscopic data.

Spectroscopic techniques are useful in food sensory analysis to measure properties such as color, texture, and flavor. The advantages of these techniques include objective measurement, non-destructiveness, and rapid results. They can provide a detailed analysis of food chemical composition, helping to identify changes due to processing or storage. However, limitations include the need for calibration and reference materials, the possibility of interference from other compounds, and the complexity of data analysis. The cost of equipment and expertise required may also be a barrier for smaller food businesses.

### 5.2. Adulteration Attributes

Adulteration refers to the practice of mixing inferior quality substances with superior original substances and or/by adding ingredients of lower quality, which can negatively impact the completeness and nutritional value of food products [217]. Often, the presence of contaminated materials is very comparable to that of the initial products, making it complicated to differentiate them when blended. Additionally, establishing the species and source of staple foods is important in protecting consumers from potential fraud, as food geographical indications cannot be confirmed solely from food labels. Thus, reliable analysis of adulteration is required to confirm the quality of the product. The scope of the proposed paragraph is to discuss the problem of food adulteration and the role of spectroscopic techniques with chemometrics in detecting and differentiating adulterated food samples. It highlights the challenges posed by adulteration, including the difficulty in differentiating contaminated materials from the original products and the importance of confirming the species and source of staple foods.

Spectroscopic techniques, such as Transmission Raman spectroscopy, have been used to achieve this goal. In a recent study, Transmission Raman spectroscopy was used to differentiate rice samples based on their geographic origin using PCA and LDA [223]. Similarly, Feng et al. (2013) used a combination of Raman spectroscopy and multivariate data analysis techniques to differentiate rice samples from various regions of China, with an overall accuracy of over 90% [224]. Different types of rice samples and their geographical origin were effectively discriminated using ^1^H-NMR spectroscopy and multivariate data analysis. The accuracy of wheat flour sample discrimination using a simple linear model was 80% [225]. The study by Esteve Agelet et al. (2012) tested the feasibility of NIR spectroscopy to discriminate viable-germinating corn and soybeans from dead seeds and found that dead corn kernels could be discriminated with an accuracy of 99% using partial least squares discriminant analysis (PLS-DA) [226], Haughey et al. (2013) used NIR spectroscopy (833–2632 nm) to detect adulteration of soybeans with melamine and achieved R^2^ values ranging from 0.89 to 0.99 using PLSR and PCR algorithms [227]. The use of Fourier transform mid-infrared (FT-MIR) spectroscopy and discriminant analysis was successful in detecting adulterated potato and sweet potato starch by Liu et al. (2013) [228]. Similarly, FT-IR spectroscopy and discriminant analysis were combined for the geographical differentiation of dried lentil seeds [229]. For imaging spectroscopy, it was examined to identify different types of wheat kernels, and found that better results were obtained by selecting three specific wavelength intervals. The ability of NIR spectroscopy to detect adulteration of soybeans by melamine was demonstrated, with R^2^ values of 0.89 to 0.99 using PLSR and PCR algorithms [230]. NIR spectroscopy was also used to distinguish viable-germinating corn and soybeans from dead seeds, with perfect accuracy based on PCA and PLS-DA [231]. The NIR and HSI in combination with PCA to detect residues of 0.10% peanuts in wheat flour, with an R^2^ of 0.95 [232]. All these spectroscopic techniques demonstrated very great accomplishments in detecting different forms of food fraud and authentication. The end points of the paragraph include the effectiveness of spectroscopic techniques in detecting and differentiating adulterated food samples based on their chemical composition. It mentions the advantages of spectroscopic techniques, such as high sensitivity, specificity, and non-destructiveness, which allow for the identification of small changes in the food samples.

Spectroscopic techniques detect food adulteration by analyzing the chemical composition of food samples. Advantages include high sensitivity, specificity, and non-destructiveness, which can identify small changes in the food sample. Limitations include the need for calibration and reference materials, interference from other compounds, limited applicability, and cost of equipment and expertise.

### 5.3. Chemical Attributes

The chemical composition of food plays a crucial role in determining its nutritional value and consumer acceptance. The scope of the proposed paragraph is to discuss the chemical composition of food, focusing on cereals and legumes, and highlight the importance of accurate measurement and control of their chemical components for determining nutritional value and food quality. The main chemical components in cereals and legumes are carbohydrates, protein, moisture, fiber, fat, and ash. Starch, a type of carbohydrate, is composed of helical amylose and branched amylopectin. The nutritional value and consumer acceptance of food are significantly influenced by its chemical composition. Cereals and legumes primarily consist of carbohydrates, protein, moisture, fibre, fat, and ash. For instance, maize can range from 20% to 36%, sorghum from 21% to 35%, wheat from 17% to 29%, barley from 11% to 26%, rice from 8% to 37%, pea from 34% to 37%, and potatoes from 18% to 23% [233]. Among these components, starch, a type of carbohydrate, is composed of amylose and amylopectin. The relative proportions of amylose and amylopectin in starch have a notable influence on the quality of food products such as bread and noodles [234]. The content of those chemicals can vary greatly which can influence the food quality properties and high-performance techniques needed to control their concentrations. Staple foods are primarily composed of proteins, which contribute to their structural and functional properties, affecting their quality and taste. Moisture content is a major factor that influences the shelf life and germination success of staple foods. The fiber in these foods, made up of cellulose, hemicellulose, lignin, pectin, and gums, can help lower cholesterol levels but does not provide energy. Lipids are the most energy-dense component, providing more energy per gram than carbohydrates and proteins. Ash content, which is the inorganic residue after organic matter is burned, indicates the presence of minerals in the food sample. Moisture content is commonly measured through drying with an oven or Karl Fisher titration, both of which have low efficiency [235]. The content of crude fat in staple foods can be measured by extracting the dried material using ether or petroleum ether. The crude protein content is typically determined using Kjeldahl or Dumas methods, which quantify the organic nitrogen content [236]. However, these traditional methods are destructive, time-consuming, and require a long preparation time. To overcome these limitations, the use of non-destructive and rapid detection methods is desired. The VIS/IR spectroscopy techniques hold the potential for determining the chemical components in staple foods. Nie et al., demonstrated that VIS/NIR spectroscopy in the range of 400 to 1000 nm can be used non-destructively to determine the presence of the poisonous phytohemagglutinin in beans by monitoring the boiling time of yard-long beans [237]. Both NIR and MIR techniques were used to verify the chemical characteristics, as well as protein, lipid, moisture, and ash amounts in soybean, while PLSR models showed good performance [238]. The preparation and determination of staple food samples using VIS/IR spectroscopy techniques take less than five minutes, compared to the 10–16 h required by traditional methods. FT-NIR spectroscopy has been shown to estimate the concentration of moisture, protein, lipid, ash, and carbohydrate in Brazilian soybeans with high accuracy [239]. The protein and moisture content had the best results with correlation coefficients of 0.81 and 0.80, respectively. NIR spectroscopy has also been used to accurately predict the crude protein content in potatoes, with high correlation coefficients ranging from 0.86 to 0.95 using PLSR for calibration [240]. The VIS/NIR (446–1125 nm) was applied for chemical components in two varieties of potato tubers [241]. The chemical and enzymatic compositions of staple foods, including phenolics, flavonoids, anthocyanins, carotenoids, dioscin, and catalase, have been screened using NIR or MIR spectroscopy and analyzed using chemometric methods namely HCA, PCA, SVM and PLS-DA, producing good predictive ability [241,242,243]. The endpoints of this section include the advantages of spectroscopic techniques in offering high sensitivity, specificity, and non-destructiveness for food chemical analysis. It mentions their ability to quickly identify small changes in food composition and measure parameters such as acidity, pH, and moisture content.

Spectroscopic techniques offer high sensitivity, specificity, and non-destructiveness for food chemical analysis. They can quickly identify small changes in food composition and measure parameters such as acidity, pH, and moisture content. However, limitations include the need for calibration and reference materials, interference from other compounds, and the complexity of data analysis. The cost of equipment and expertise required may also be a barrier for smaller food businesses.

### 5.4. Mycotoxin Attributes

The presence of mold and its associated toxins during post-harvest storage can result in a decrease in food quality, leading to losses in nutrients and market value, as well as pose serious food safety risks. The scope of the proposed paragraph is to discuss the presence of mold and mycotoxins in post-harvest storage and their negative impact on food quality, safety, and market value. It emphasizes the harmful effects of mycotoxins, such as aflatoxins and Fusarium toxins, which are known to be carcinogenic and linked to liver and lung cancer in humans.

Mycotoxins, like aflatoxins and Fusarium toxins, are harmful byproducts produced by mold and are recognized as carcinogenic, linked to liver and lung cancer in humans. According to estimates, as much as 25% of crops grown for both animal feed and human consumption globally may be contaminated with mycotoxins [244]. Mycotoxin amounts for essential food products have been specifically restricted by the EU [245]. In the USA, 20 ppb (parts per billion) of aflatoxin amounts in food and 100 ppb in feed are permitted for the administrative market [246]. Giving to the FAO, one billion metric tons of food are rotten worldwide yearly caused by mycotoxins [247]. Hence, the ability to accurately detect various levels of fungal contamination can greatly aid in controlling plant diseases and reducing food safety hazards. Currently, the methods for identifying and measuring toxins consist primarily of thin-layer chromatography and high-performance liquid chromatography, but these methods are both expensive and time-consuming and involve the destruction of samples [248]. The detection of mycotoxins in staple foods can be performed quickly and easily using IR spectroscopy. The NIR spectroscopy in the range of 950–1650 nm was used in conjunction with PLSR to detect total fungal and yellow-green Aspergillus flavus infections in rice [249]. However, the accuracy levels for both total fungi and yellow-green A. flavus infections were not high. The PLS was based on full cross-validation to detect fumonisin contamination in maize through NIR spectroscopy, achieving a high R^2^ of 0.91. This demonstrates that NIR spectroscopy is a viable alternative tool for detecting fumonisin infections [250]. The FT-MIR spectroscopy in the range of 2500–16,000 nm with an attenuated total reflectance unit to differentiate peanut kernels contaminated with aflatoxin and non-aflatoxin strains. The “Acceptable” stream (aflatoxin ≤ 20 ppb) was separated from “Mildly” (20 < aflatoxin < 300 ppb), “Highly Toxic” (300 < aflatoxin < 1200 ppb), and “Highly Moldy” (aflatoxin > 1200 ppb) through classification. The fingerprint region (5556–12,500 nm) was utilized to predict the A. flavus and A. parasiticus species with varying levels of contamination based on PLS regression, achieving an R^2^ of 99.98% [251]. The utilization of the Raman technique in combination with PCA allows for the non-invasive detection of deoxynivalenol (commonly known as vomitoxin) in contaminated wheat and barley [252]. The benefits of this approach consist of the utilization of a NIR laser excitation (1064 nm), which minimized disruption from the fluorescence of biological substances. The endpoints of the paragraph highlight the advantages of spectroscopic techniques in food mycotoxin analysis, including high sensitivity, specificity, and non-destructiveness. It emphasizes the ability of these techniques with chemometric models to detect even small amounts of mycotoxins in food samples and provide rapid measurements of various chemical parameters.

Spectroscopic techniques offer advantages in food mycotoxin analysis such as high sensitivity, specificity, and non-destructiveness. These techniques can detect even small amounts of mycotoxins in food samples and provide rapid measurements of various chemical parameters. However, limitations include the need for calibration and reference materials, the interference of other compounds in the sample, and the limited applicability of certain techniques to specific mycotoxins.

### 5.5. Parasitic Contamination

Staple foods can be degraded and lose market value due to contamination from parasitic insects. The scope here is to discuss the contamination of staple foods by parasitic insects and the detrimental effects they have on food quality and market value. The activities of parasitic insects, both external and internal, and their impact are highlighted.

The insects not only feed directly on the food, but also create heat and moisture through their metabolic activity, leading to localized hotspots and spoilage. This results in weight loss, nutrient depletion, reduced germination ability, and increased risk of contamination during storage. External insects like the Oryzaephilus surinamensis and internal insects such as Sitophilus granarius can both harm stored products. Some insects like the sweet potato weevil (Cylas formicarius elegantulus) do most of the damage inside the food without significant external changes. Others, like the rice weevil (Sitophilus oryzae) and lesser grain borer (Rhyzopertha dominica), lay eggs, larvae, or pupae in seeds, continuing their destructive activity for up to 7 weeks, until the adult insects emerge and leave an exit hole, making the damage visible [253].

The detection of parasitic insect infestations in staple foods is crucial for the food industry. Such infestations can decrease the quality and value of the food. Internal infestations are more challenging to detect and require effective methods for inspection. HSI, a spectral information technique, has the potential to provide information about internal infestations through reflectance or absorbance measurements. A study by Singh et al. (2009) used LW-NIR HSI (900–1700 nm) to differentiate between insect-infested and healthy wheat kernels, achieving an accuracy of over 85% with LDA and QDA classifiers [254]. The endpoints of the paragraph emphasize the importance of detecting parasitic insect infestations in staple foods to mitigate the negative effects on food quality and value. Internal infestations are highlighted as being more challenging to detect and requiring effective inspection methods.

Spectroscopic techniques detect parasitic contamination in food with high sensitivity, specificity, and non-destructiveness. These techniques rapidly and precisely measure various chemical parameters, but limitations include the need for calibration and reference materials, the possibility of inaccurate results due to other compounds in the sample, and limitations in detectability for certain parasites. The cost and expertise required may also be a barrier for smaller food applications.

### 5.6. Internal Functional Characteristics

Functional disturbances within staple crop plants are linked to irregular growth patterns brought on by less-than-ideal environmental factors. The scope here is to discuss briefly some examples of the internal functional characteristics disturbances within staple crop plants such as those caused by environmental factors.

The functional disturbances include variations in temperature, moisture, oxygen, nutrients, the presence of toxic gases, and a deficiency in growth regulators. The symptoms of these internal conditions appear to be root tuber disorders such as hollow heart, black heart, and internal brown spot. Overuse of nitrogen fertilizer during the growth phase can cause hollow heart in tubers, a condition where the tuber’s core dies or splits, creating a cavity [255]. This disorder is commonly triggered by the rapid growth of the tuber and results in reduced storage life, poorer quality chips, and an unattractive appearance. Blackheart is a condition that arises when there’s insufficient oxygen during the tuber’s growth or storage period. Tubers cultivated in excessively damp areas or those exposed to severe temperatures are more prone to this condition. Internal brown spot refers to the internal death of the tuber’s central tissue, which significantly diminishes its culinary worth. Other physiological damage, such as freezing or chilling injuries, occurs due to extended exposure to freezing temperatures post-harvest. The symptoms of these injuries manifest as grey or black patches, or a brown discoloration around the tuber’s vascular ring. Even though these internal physiological disorders may not be immediately visible, they greatly affect the quality and value of root and tuber crops. X-ray examination has been reported to successfully detect hollow hearts in potatoes but attempts to use acoustic methods for detection have been unsuccessful [256], although unsuccessful using an acoustics method [257]. Traditional methods are also heavily dependent on the orientation of the potato. However, spectroscopic techniques have been shown to be effective for the non-destructive prediction of internal physiological disorders, including black heart, hollow heart, and internal brown spot in root tubers. The VIS/NIR transmission spectroscopy (513–850 nm) used to compare three different morphological correction methods combined with PLS-DA and PCA to detect black hearts in potatoes. The best performance was found with height-corrected transmittance. Using six wavelengths (839, 817, 741, 711, 698 and 678 nm), the overall validation classification rate for the black heart was 96.53% [258]. The HSI in the 1000–1700 nm range was applied to distinguish hollow heart and internal brown spots in tubers. Specifically, the HSI technique was applied to detect the presence of hollow hearts in potato tubers [259]. An accurate recognition rate of 89.10% was achieved by combining support vector machine (SVM) with various image processing techniques. Similarly, time-resolved reflectance spectroscopy (540–900 nm) was used for non-destructive external measurement of internal brown spots in potato tubers [260]. By internal detection of healthy tissue and black spots, the most sensitive wavelength for detection was found to be 690 nm. The endpoints of the paragraph emphasize the negative effects of these internal physiological disorders on the quality and value of root and tuber crops.

Spectroscopic techniques offer insights into food’s internal functional characteristics, such as composition, structure, and functional properties, with high sensitivity, specificity, and non-destructiveness. They allow for rapid and precise measurements of various chemical and physical parameters. However, challenges arise from the need for calibration and reference materials, potential interference from other compounds, and limitations in the applicability of certain techniques. Moreover, the cost of equipment and expertise required may be an obstacle.

## 6. Conclusions

In conclusion, advancing spectroscopy techniques offer a game-changing solution for food composition analysis, presenting a non-destructive, rapid, cost-effective, and eco-friendly alternative to traditional methods. These techniques provide valuable insights into food quality, chemical components, composition, structure-function relationships, and sensory attributes, eliminating the need for extensive sample preparation in many cases, thus saving time and resources. Coupled with appropriate chemometric multivariate methods, spectroscopy enables comprehensive and accurate analyses of various food materials.

This review has highlighted recent studies showcasing the effectiveness of spectroscopy techniques, such as hyperspectral and multispectral imaging, NMR, IR, Raman, X-ray-based methods, fluorescence, and UV-visible, in conjunction with chemometric approaches for diverse food analysis applications. From determining the chemical composition and identifying geographical origin to ensuring food safety and traceability, monitoring storage and preservation, assessing sensory characteristics, evaluating microbial quality, and detecting food spoilage, these techniques have shown immense potential and versatility in the food industry.

Looking ahead, the future of spectroscopic techniques in food analysis appears highly promising. As technology continues to advance, these techniques are expected to become even more powerful, accurate, and efficient, opening new avenues of research and application. It is strongly recommended that food businesses and researchers embrace spectroscopy in their analyses and consider integrating these techniques into their standard practices to enhance the overall efficiency and quality of food analysis.

However, it is crucial to acknowledge the challenges and limitations that may arise with spectroscopic methods, such as issues related to sample heterogeneity, sensitivity, accuracy, and the need for proper calibration and reference materials. Chemometric methods, while invaluable, require careful handling to avoid issues like overfitting and to ensure reliable results, potential drawbacks of chemometric methods, such as overfitting or the need for extensive data processing and model optimization should be also considered.

As the field progresses, further research and development are essential to optimize and expand the capabilities of spectroscopic techniques in food analysis. By addressing these challenges and pushing the boundaries of innovation, spectroscopy can revolutionize the food industry, bolstering food safety, quality control, and product innovation in a sustainable and impactful manner.

## Figures and Tables

**Table 1:** Advanced spectroscopic techniques combined with chemometric approaches for quantitative analysis in food application. Table was ordered regarding used techniques.

Quantitative Analysis						
Authors	Food Materials	Fingerprinting Technique	Analysed Components	Objectives	Chemometric Analysis	Ref.
Gamela et al.	Cocoa beans	EDXRF	Cu, K, Sr and Zn	Cocoa beans	PLS	[56]
Sperança et al.	Bananas	X-ray fluorescence	Zn	Determination Zn content in Banans	PLS	[57]
Priyashantha et al.	Cheese	Near-infrared hyperspectral (NIR-HS) imaging	-	Explain the relationship between average spectra and cheese maturity	PLS	[58]
Darnay et al.	Semi-hard cheese	HSI	Transglutaminase	Detection of the enzyme of transglutaminase in the cheese	PLS	[59]
Lu et al.	Potatoes	Fluorescence HSI	Solanine	Predict the solanine content in potatoes	SVR	[60]
Xiao et al.	Fresh-cut potato	HSI	Color parameters (bruising index) and water content	Assess the quality of potatoes	LS-SVM and PLS	[61]
Tian et al.	Purple sweet potato	Vis-NIR HSI	Moisture and anthocyanins	Predict the critical indexes of moisture and anthocyanins in purple sweet potato	PLSR	[62]
Li et al.	Plum	VNI-HSI	Nan	Predict the soluble solid contents and the color of two plums cultivars	PLS	[63]
He et al.	Wheat flour	HSI	Talcum powder	Detection of talcum powder adulterated in wheat flour	SNV-CARS-PLS	[64]
Kim et al.	Wheat flour	SWIR-HSI	Benzoyl peroxide	Detecting the bleaching agent of benzoyle peroxide in wheat flour	PLS	[65]
Sun et al.	Melons	HSI	Nan	Predict the sugariness and hardness of melons	PLS, SVM and ANN	[66]
Wang et al.	Chinese steamed bread	FT-NIR	Potato flour content	Predicting potato flour in Chinese steamed bread	PLS-R	[67]
Tu et al.	Wheat flour	FT-NIR	Talcum powder	Quantitation of low content of talcum powder in wheat flour	Gradient-boosted decision tree (GBDT)	[68]
Kandpal	Tuber flour	NIR and MIR	Chemical components:amylose, starch, protein, glucose, cellulose, and moisture contents	Prediction of quality traits in tuber traits by mean of Data fusion of FT-IR and FT-NIR	SOPLS	[69]
Kamboj et al.	Wheat	FT-NIR	Crude protein and carbohydrate	Compare chemometrics for predicting the quality parameters of wheat	PLS, MLR, SVM	[70]
Liang et al.	Potatoes	FT-NIR	Sugar content	Detection of zebra chip disease (ZC) in potatoes	PLS	[71]
Jiang et al.	Wheat flour	Portable NIR	Fatty acid	Quantitation of fatty acids in wheat	Variable combination population analysis (VCPA), extreme learning machine (ELM)	[72]
Ning et al.	Wheat grains	FT-NIR	Zearalenone	Detection of zearalenone in wheat	SVM	[73]
Cámara et al.	Clove and pomegranate	IR	Antioxidant activity	Estimation of antioxidant activity in clove and pomegranate	MCR-ALS and PLS	[74]
Castro et al.	Peanut oil	NIR and Raman	Adulterants (corn oil and vegetable oil)	Assessment of vibrational spectroscopy with chemometrics	MCR-ALS and PLS regression	[75]
Castro et al.	Saffron	FT-NIR	Saffron adulterants (onion, calendula, pomegranate, and turmeric)	Detection of Saffron adulterants	MCR-ALS and PLS regression	[76]
Li et al.	Saffron	FT-NIR	Saffron adulterants (lotus stamens and corn stigmas)	Detection of saffron adulterants	Synergistic interval PLS (SI-PLS), competitive adaptive reweighted sampling PLS (CARS-PLS)	[77]
Li et al.	Saffron	FT-NIR	Corcin	Determination of corcin content in Saffron	PLS	[78]
Liu et al.	Panax notoginseng	FT-NIR	Adulterants (hizoma curcumae, *Curcuma longa* and rhizoma alpiniae offcinarum)	Quantification of Panax notoginseng with its adulterants	PCR, PLS, ELM and SVR	[79]
Liu et al.	Vegetable oils	FT-NIR	Phytosterols	Determination of phytosterols in vegetable oils	Pls	[80]
Joshi et al.	Eggs	FTIR	Constituents of eggs (yolk and albumen)	Detection of fabricated eggs	PLS-DA and SVM	[81]
Mazivila et al.	Milk	FT-NIR	Melamine and sucrose	Estimation the adulterant contents in the milk	MCR-ALS	[82]
Novianty et al.	Palm fruit	FT-NIR	Oil content	Quantitation of oil content in palm fruit	EMD-ANN	[83]
Basar et al.	Honey	FTIR	Adulterant (beet sugar and corn syrup)	Determination of honey adulteration	Genetic-algorithm-based inverse least squares (GILS) and (PLS)	[84]
Qin et al.	Wheat flour	Raman chemical imaging	Benzoyl peroxide	Detection of benzoyle peroxide	PLS	[85]
Yuan et al.	Duck meat	Surface-enhanced Raman	Testosterone propionate and nandrolone residues	Quantitation of residues in the duck meat	LS-SVR	[86]
Nakajima et al.	Banana	Raman	Starch	Quantification of starch in banana	PLS	[87]
Hara et al.	Tomatoes	Raman	Carotenoids	Determination of carotenoids in tomatoes	PLS	[88]
De Olieveira mendes et al.	Raw milk	Raman	Whey	Quantitation of whey in raw milk	PLS	[89]
Czaja et al.	Youghurts	Raman	Fat, lactose, and protein	Determination of nutritional parameters of yoghurts	PCA and PLS	[90]
Tian et al.	Milk	Raman	Adulterants (maltodextrin, sodium carbonate, and whey)	Prediction of adulterants in raw milk	PLS	[91]
Berzins et al.	Breast milk	Raman and FTIR	Macronutrients (protein, fat, and carbohydrate)	Determination of macronutrients in the breast milk	PLS	[92]
De sa oliveira et al.	Spreadable cheese	Raman	Starch	Quantitation of starch in adulterated spreadable cheese	PLS	[93]
Liu et al.	Edible oils	Raman and FT-IR data fusion	Peroxide values and acid values	Determination of chemical quality indices of edible oils during thermal oxidation	PLS	[89]
Puertas et al.	Egg yolk	Data fusion of FTIR and UV-Vis	Cholesterol	Prediction of cholesterol in egg yolk	PLS and PCR	[94]
Wang et al.	Infant formula	Vis-NIR and Raman data fusion	-	Assessment of infant formula storage temperature and time	SVM	[95]
Valinger et al.	Honey	UV-Vis and NIR data fusion	Sugar syrups	Detection of honey adulteration	PLS and ANN	[96]
Wang et al.	Camelia oil	Excitation-emission matrix fluorescence	Vegetable oils	Quantitation of adulterant in camelia oil	N-PLS and PARAFAC	[97]
Baretto et al.	Milk	Fluorescence	Melamine	Determination of melamine in milk	PARAFAC and UPLS	[98]
Gu et al.	Rapessed oil in water	Fluorescence	Lipid	Quantitative assessment of lipid oxidation in a rapeseed oil-in-water	GA-SVR	[99]
Tarhan	Extra virgin olive oil (EVOO)	FTIR, UV–Vis and fluorescence	Squalene	Quantification of squalene in extra virgin olive oils	PLS	[100]
Wu et al.	Edible blend oil	UV-Vis	Adulterant (vegetable oil)	Quantification of vegetable oils in edible blend oil	Weighted multiscale SVR	[101]
Zhang et al.	Edible oils	UV-Vis	Acid value	Impact of heating on edible oils	PLS and PCR	[102]
Rios-Reina et al.	Wine and balsamic vinegar	UV-Vis	Grape-must caramel (E-150d caramel)	Quantitation of grape-must caramel in wine and balsamic vinegars	PLS	[103]
Cavdaroglu et al.	Vinegar	UV-Vis and MIR	Phenolic components, p-coumaric and syringic acids, citric and acetic acids,	Predict quality and chemical parameters of vinegar	PLS and OPLS	[104]
Santos et al.	Milk	NMR	Adulterants (Whey, urea, hydrogen peroxide, synthetic urine and synthetic milk)	Quantification of milk adulteration	PLS	[105]
Liu et al.	Cream	NMR	Artificial bright blue pigment	Detecting additives content in cream	PLS and MLR	[106]
Sun et al.	Carrot, banana and pleurotus eryngii	NMR	Moisture	Monitor water states of typical fruits and vegetables during microwave vacuum drying	PLS, SVM and BP-ANN	[107]
Hajjar et al.	Hen egg	NMR	Fatty acids	Quantification of fatty acids in hen eggs	PLS	[108]
Galvan et al.	Edible oils	NMR	Fatty acids and iodine value	Analysis of edible oils	PLS and SVR	[109]
Haddad et al.	Cheese	NMR	Fatty acids	Quantitation of individual fatty acids	PLS	[110]
Jiang et al.	Rice	Surface-enhanced Raman scattering	Chlorpyrifos residue	Quantify chlorpyrifos residues in rice samples	GA-PLS, UVE-PLS, VCPA-PLS and CARS-PLS	[111]
Richardson et al.	Coconut water	Raman	Sugars	Detection of adulteration in Coconut water	PLS	[112]

**Table 2:** Advanced spectroscopic techniques combined with chemometric approaches for qualitative analysis in food application. Table was ordered regarding used techniques.

Qualitative Analysis						
Authors	Food Materials	Fingerprinting Technique	Analysed Components	Objectives	Chemometric Analysis	Ref.
Galvan et al.	Tomato and sweet paper	EDXRF	-	Discrimination of tomato or sweet pepper samples effectively according to the agronomic production mode or geographical origin	PLS-DA	[113]
Scatigno et al.	EVOO	EDXRF	Ni, Fe and Ti	Discrimination of EVOO	PCA	[114]
Panebianco et al.	Tomato fruit	XRF	-	Establish an assessment procedure for the origin and quality assessment of Sicilian tomato fruits	PCA	[115]
Allegretta	Beans	TXRF	-	Clustering of the seeds of beans according to their geographical origin	PCA and PLS-DA	[116]
Vitali et al.	Croatian wines	TXRF	Contents of metals (K, Ca, Fe, Cu, Zn, Mn, Sr, Rb, Ba, Pb, Ni, Cr and V)	Classification of origin and type of Croatian wines	PCA and cluster analysis	[117]
Li et al.	Peaches	Short-wave near-infrared (SW-NIR) and long-wave near-infrared (LW-NIR) hyperspectral imaging	-	Detection bruises in peaches	PCA	[118]
He et al.	Flour	Vis-NIR HSI	Mites *Tyrophagus putrescentiae* and *Cheyletus eruditus*	Detection of mites *Tyrophagus putrescentiae* and *Cheyletus eruditus* in flour	Random forest and PCA-ANN	[119]
Al-Sarayreh et al.	Meat	NIR-Vis HSI	-	Deep learning approach for red-meat classification by combining the spectral and spatial features of HSI data	CNN	[120]
Pan et al.	Peaches	Hyperspectral reflectance imaging	-	Detection of cold injury in peaches	ANN	[121]
Sun et al.	Peaches	Hyperspectral reflectance imaging	-	Characterization of chilling injury in peaches	PLS-DA, ANN and SVM	[122]
Babellahi et al.	Green bell peppers	HSI	-	Detection of chilling injury in green bell peppers	PLS-DA	[123]
Cen et al.	Cucumber fruit	HSI	-	Detection of chilling injury in cucumber fruit	SVM and KNN	[124]
Carreiro Soares et al.	Cotton seeds	HSI	-	Discrimination of different varieties of seeds	PLS-DA	[125]
Fan et al.	Blueberry	HSI	-	Detection of blueberry internal bruising over time	LS-SVM	[126]
Sun et al.	Tomatoes	HSI	-	Characterization of bruised tomatoes	PLS-DA	[127]
Susic et al.	Tomatoes	HSI	-	Discrimination between abiotic and biotic drought stress in tomatoes	PLS-DA PLS-SVM	[128]
Zhao et al.	Wheat seeds	HSI	-	Characterization the purity of wheat seeds	CNN	[129]
Zhao et al.	Maize seeds	HSI	-	Classification of maize seeds	Neural network	[130]
Tsouvaltzis et al.	Eggplant fruit	FT-NIR and NIR-HSI	-	Evaluating the temperature effect on chilling injury of eggplant	PLS-DA, SVM and KNN	[131]
Liang et al.	Potatoes	FT-NIR	Sucrose, glucose fructose	Detection of zebra chip disease (ZC) in potatoes	Canonical discriminant analysis	[71]
Huang et al.	Honey	NIR and FTIR	Syrup adulterant	Distinguish the normal honey from adulterant one	SVM	[132]
De Girolamo	Wheat	FT-MIR and FT-NIR	Ochratoxin A	Assessment of the adulteration of wheat by ochratoxin	PLS-DA and PC-LDA	[133]
Chen et al.	Eggs	FT-NIR	-	Verifying the authenticity of native eggs	Data-driven-based class-modeling (DDCM), PCA	[134]
Liu et al.	Panax notoginseng	FT-NIR	Adulterants (rhizoma curcuma, *Curcuma longa* and rhizoma alpiniae offcinarum)	Identification of panax notoginseng with its adulterants	PLS-DA and SVM	[79]
Marquetti et al.	Arabica Coffee	FT-NIR	-	Evaluation of geographic and genotypic origin of arabica coffee	PLS-DA	[135]
Mazivila et al.	Milk	FT-NIR	Melamine and sucrose	Discrimination of pure milk from the adulterant one	DD-SIMCA	[82]
Miao et al.	Rice	FT-NIR	-	Classification of rice based on storage time	PCA, KNN and PLS-DA	[136]
Rovira et al.	Cashew nuts	FT-NIR	Adulterants (peanuts)	Characterization of the adulterant cashew nuts by other nuts	SIMCA	[137]
Visconti et al.	Cheese	FT-NIR	Cellulose and silicon dioxide	Determination of additives in the grated hard cheese	PLS-DA	[138]
Xie et al.	Waxy rice	FT-NIR	Amylose and amylopectin	Determination of quality parameters by FT-NIR	Modified PLS (MPLS)	[139]
Ziegler et al.	Kernels and flours	FT-NIR	-	Differentiation of flours and kernels of costly ancient species from less expensive bread wheat	PLS-DA	[140]
Joshi et al.	Eggs	FTIR	Constituents of eggs (yolk and albumen)	Detection of fabricated eggs	PLS-DA and SVM	[81]
Rozali et al.	Crude palm oil	FTIR	-	Authentication of different geographical and temporal origins of crude palm oils	OPLS-DA	[141]
Li et al.	Hazelnuts	FT-Raman and NIR data fusion	Almonds adulterant	Discriminate the unadulterated hazelnuts from the adulterated hazelnuts with almonds	SIMCA	[142]
Yuan et al.	Duck meat	Surface-enhanced Raman	Testosterone propionate and nandrolone residues	Classification of duck meat based on residues	Particle swarm optimization–support vector classification (PSO-SVC)	[86]
Unuvar et al.	Durum wheat flour	Raman spectroscopy, FT-NIR, synchronous fluorescence spectroscopy (SFS), (ATR-FTIR)	-	Distinguishing common and durum wheat flour samples with different genotypes	PCA, PLS-DA	[143]
Amjad et al.	Milk	Raman	Proteins, milk fats, lactose	Differentiation between milk samples of different species	Random forest classifier (RF), PCA	[144]
De Oliveira et al.	Enriched eggs	Raman	Omega-3 fatty acids	Discrimination between conventional and omega-3-fatty acids enriched eggs	PLS-DA	[145]
De sa oliveira et al.	Spreadable cheese	Raman	Starch	Classify spreadable cheese as adulterated or without starch	PLS-DA	[93]
Nieuwoudt et al.	Milk	Raman spectroscopy	Nitrogen-rich molecules and sucrose	Detecting adulteration of milk	PLS-DA	[146]
Ning et al.	Duck meat	Raman	Sulfadimidine and Sulphapyridine	Classification of duck meat based on Sulfadimidine and Sulphapyridine	SVM and PCA	[87]
Robert et al.	Meat	Raman	-	Discrimination between different species of meat (intact beef, venison, and lamb meat)	PLS-DA	[147]
Tian et al.	Milk	Raman spectroscopy	Adulterants ofaltodextrin, sodium carbonate, and whey	Distinguishing raw milk from the adulterated one	PLS-DA	[91]
Tian et al.	Rice	Raman spectroscopy	-	Distinguishing rice based on producing areas	PCA-KNN, SPA-KNN, PCA-LS-SVM and SPA-LS-SVM	[148]
Wu et al.	Honey	Raman spectroscopy	Adulterants (fructose corn syrup, rice syrup, maltose syrup, blended syrup)	Characterization of adulterant honey	CNN	[149]
Wang et al.	Infant formula	Vis-NIR and Raman data fusion	-	Assessment of infant formula storage temperature and time	SVM	[95]
Yao et al.	Boletus mushrooms	Data fusion of FT-IR and UV	-	Discrimination of different geographical origins of Boletus mushrooms	PLS-DA and SVM	[150]
Antonio et al.	Honey	Spectrofluorimetry	Adulterants (corn syrup, sugar cane molasses and polyfloral honey)	Detection of adulterations in a valuable Brazilian honey	Multilinear PLS-DA (NPLS-DA), unfolded PLS-DA (UPLS-DA), PARAFAC	[151]
Fang et al.	Chinese lager beers	Excitation-emission matrix fluorescence	-	Characterization and classification of Chinese pale lager beers produced by different manufacturers	PARAFAC-KNN	[152]
Jiménez-Carvelo et al.	Extra virgin olive oils	Fluorescence and NIR	Adulterant (vegetable oil)	Authenticate the geographic origin of Argentinean EVOO samples	NPLS–DA	[153]
Meng et al.	Olive oil	Excitation-emission matrix fluorescence	Adulterant (soybean)	Detection of adulteration of olive oil with soybean oil	Multiway-PCA (MPCA), ANN, PLS-DA	[154]
Yuan et al.	Edible vegetable oils	Infrared, NIR and fluorescence	-	Identification of different vegetable oils	MPCA, NPLS-DA	[155]
Uncu et al.	Fresh olive oils	Mid-infrared, UV–Vis and fluorescence	Adulterant (old olive oil)	Detection of adulteration of olive oil	OPLS-DA	[156]
Gonçalves et al.	Monovarietal Extra Virgin Olive Oils	UV-Vis	Phenolic compounds	Monitor the behavior of autoxidative processes through the storage time in two packaging systems of different EVOO	MCR-ALS	[157]
Suhandy et al.	Peaberry coffee	UV-Vis	-	Classify coffee samples as either pure peaberry or pure normal coffee	SIMCA and PLS-DA	[158]
Torrecilla	Vinegar	UV-Vis	-	Characterization of vinegars produced from six different raw materials	PLS-DA and ANN	[159]
Cavdaroglu et al.	Vinegar	UV-vis and FTIR	Adulterant (spirit vinegar and acetic acid)	Discrimination of non-adulterated vinegar from the adulterated	ANN	[160]
Kucharska-Ambrożej et al.	Mint	UV-Vis and FTIR		Distinguish between two species of mint (peppermint or spearmint)	PLS-DA and SVM	[161]
Botoran et al.	Fruits	NMR	Amino acid	Differentiation of the fruit samples in varietal origin	PCA and LDA	[162]
Consonni et al.	Coffee	NMR	Fatty acids, β-(1-3)-d-galactopyranose, quinic acid and its cyclic ester)	Characterizing organic roasted coffee from the conventional roasted coffee	OPLS-DA	[163]
De Moura Ribeiro et al.	Roasted coffee	NMR	Adulterants (corn, coffee husks, barley, and soybean)	Investigating the authenticity of the roasted coffee	PCA	[164]
Da Silva et al.	Larger beer	NMR	Carbohydrates	Discriminate lager beer samples from two different classes, according to their style and information provided on the label	PCA, PLS-DA	[165]
Gougeon et al.	Wines	NMR	-	Classifying wines of different geographical origins	OSC-PLS-DA	[166]
Marseglia et al.	Cocoa beans	NMR	Amino acids, polyalcohols, organic acids, sugars, methylxanthines, lipids	Assess the geographical origin of cocoa beans	OSC-PCA, OPLS-DA	[167]
Milani	Ground coffee	NMR	Adulterants	Authentication of roasted and ground coffee based on adulterants	PCA, SIMCA	[168]
Rachineni et al.	Honey	NMR	Adulterants (brown rice syrup, corn syrup, and jaggery syrup)	Identifying type of sugar adulterants in honey	Deep learning-based neural network	[169]
Santos et al.	Milk	NMR	Adulterants (Whey, urea, hydrogen peroxide, synthetic urine and synthetic milk)	Detection of adulterated milk	SIMCA, KNN	[105]
Shi et al.	Camelia oils	NMR	Adulterants (cheap vegetable oils)	Detection of adulteration in camellia oils	PCA, OPLS-DA	[170]
Zhang et al.	Edible oils	NMR	Fatty acids	Distinguishing plant origin of edible oils	PCA, OPLS-DA	[171]

## Data Availability

The data used to support the findings of this study can be made available by the corresponding author upon request.
